# Aorta Regulatory T Cells with a Tissue‐Specific Phenotype and Function Promote Tissue Repair through Tff1 in Abdominal Aortic Aneurysms

**DOI:** 10.1002/advs.202104338

**Published:** 2022-01-24

**Authors:** Jingyong Li, Ni Xia, Dan Li, Shuang Wen, Shirui Qian, Yuzhi Lu, Muyang Gu, Tingting Tang, Jiao Jiao, Bingjie Lv, Shaofang Nie, Desheng Hu, Yuhua Liao, Xiangping Yang, Guoping Shi, Xiang Cheng

**Affiliations:** ^1^ Department of Cardiology Union Hospital Tongji Medical College Huazhong University of Science and Technology Wuhan 430022 China; ^2^ Key Laboratory for Biological Targeted Therapy of Education Ministry and Hubei Province Union Hospital Tongji Medical College Huazhong University of Science and Technology Wuhan 430022 China; ^3^ Department of Integrated Traditional Chinese and Western Medicine Union Hospital Tongji Medical College Huazhong University of Science and Technology Wuhan 430022 China; ^4^ Institute of Hematology Union Hospital Tongji Medical College Huazhong University of Science and Technology Wuhan 430022 China; ^5^ School of Basic Medicine Tongji Medical College Huazhong University of Science and Technology Wuhan 430030 China; ^6^ Department of Medicine Brigham and Women's Hospital and Harvard Medical School Boston MA 02115 USA

**Keywords:** abdominal aortic aneurysm, tissue‐specific, trefoil factor 1, Tregs

## Abstract

In addition to maintaining immune tolerance, Foxp3^+^ regulatory T cells (Tregs) perform specialized functions in tissue homeostasis and remodeling. However, whether Tregs in aortic aneurysms have a tissue‐specific phenotype and function is unclear. Here, a special group of Tregs that potentially inhibit abdominal aortic aneurysm (AAA) progression are identified and functionally characterized. Aortic Tregs gradually increase during the process of AAA and are mainly recruited from peripheral circulation. Single‐cell TCR sequencing and bulk RNA sequencing demonstrate their unique phenotype and highly expressed trefoil factor 1 (Tff1). Foxp3^cre/cre^Tff1^flox/flox^ mice are used to clarify the role of Tff1 in AAA, suggesting that aortic Tregs secrete Tff1 to regulate smooth muscle cell (SMC) survival. In vitro experiments confirm that Tff1 inhibits SMC apoptosis through the extracellular signal‐regulated kinase (ERK) 1/2 pathway. The findings reveal a tissue‐specific phenotype and function of aortic Tregs and may provide a promising and novel approach for the prevention of AAA.

## Graphical abstract

A group of Tregs with a tissue‐specific phenotype and function potentially protects abdominal aortic aneurysm progression via Tff1.

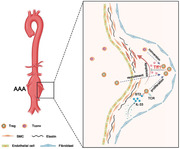

## 1 Introduction

At present, the pathogenesis of abdominal aortic aneurysm (AAA) is not fully understood. Previous studies have suggested that chronic inflammation plays an important role in the development of AAA.^[^ ^1^ ^]^ Due to atherosclerosis, haemodynamic changes, heredity, physical/chemical and other factors, the local vascular extracellular matrix becomes damaged, which is followed by inflammatory cell infiltration to remove necrotic substances and participate in tissue repair. However, inflammatory cells secrete proinflammatory cytokines during chronic inflammation to promote smooth muscle cell (SMC) apoptosis. Furthermore, inflammatory cells directly secrete cathepsins and matrix metalloproteinases (MMPs), or they indirectly promote SMC synthesis of these factors, causing extracellular matrix degradation. The result is the formation of a vicious cycle of destruction and vasodilation of inflamed tissue. Therefore, the local appearance of AAA is the result of chronic uncontrollable inflammation, and an in‐depth understanding of the local inflammatory response is essential for breaking this vicious cycle and even identifying novel therapeutic targets.^[^ ^2^ ^]^

Regulatory T cells (Tregs, defined as CD4^+^Foxp3^+^), which play an important role in the regulation of the inflammatory response and the maintenance of immune homeostasis, are a special subtype of T cells. Previous studies have suggested that Tregs are involved in the regulation of atherosclerosis,^[^ ^3^ ^]^ myocardial ischemia reperfusion injury^[^ ^4^ ^]^ and myocardial infarction.^[^ ^5^ ^]^ However, thus far, the role of Tregs in the regulation of AAA is not fully understood. Shi et al. recently discovered that in angiotensin II‐induced AAA, the transfer of Tregs reduced vascular inflammation and delayed the process of AAA;^[^ ^6^ ^]^ otherwise, removing Tregs increased tissue inflammation and aggravated AAA.^[^ ^7^ ^]^ These studies suggest that Tregs are involved in the regulation of chronic inflammation in AAA and are likely to be a potential target for treatment of AAA.

Previous studies have focused on the role of lymphoid Tregs in physiological/pathological conditions. Recently, with the progression of techniques related to immune cell isolation and analysis from tissue, tissue Tregs have received increasing attention. It has been proposed that Tregs serve multiple functions beyond their initially characterized role in immunoregulation, and an emerging body of literature suggests that tissue‐resident Tregs have specialized functions that are unique to the tissues in which they reside, such as visceral adipose tissue Tregs,^[^ ^8^ ^]^ muscle Tregs,^[^ ^9^ ^]^ lung Tregs,^[^ ^10^, ^11^ ^]^ skin Tregs,^[^ ^12^ ^]^ brain Tregs,^[^ ^13^ ^]^ and heart Tregs.^[^ ^14^ ^]^ Compared to Tregs of lymphoid organs, tissue Tregs have the following unique characteristics:^[^ ^15^ ^]^ accumulation in the tissue; different sources and maintenance mechanisms; and tissue‐specific phenotype and functional characteristics. In the present study, we found a special group of Tregs by flow cytometry and conducted a series of experiments to explore their features and functions in AAA. The results showed that this group of unique tissue Tregs, namely, aorta Tregs, was characterized by a distinct phenotype, source, maintenance mechanism and expression of functional molecules. Unexpectedly, we found that trefoil factor 1 (Tff1), a molecule that has not received attention in cardiovascular diseases in the past, can inhibit the apoptosis of SMCs and promote vascular repair. Our research may provide new insights and mechanisms for clinical intervention in AAA.

## 2 Results

### 2.1 A High Proportion of Tregs Infiltrate the Aorta During AAA Progression

**Figure 1:**
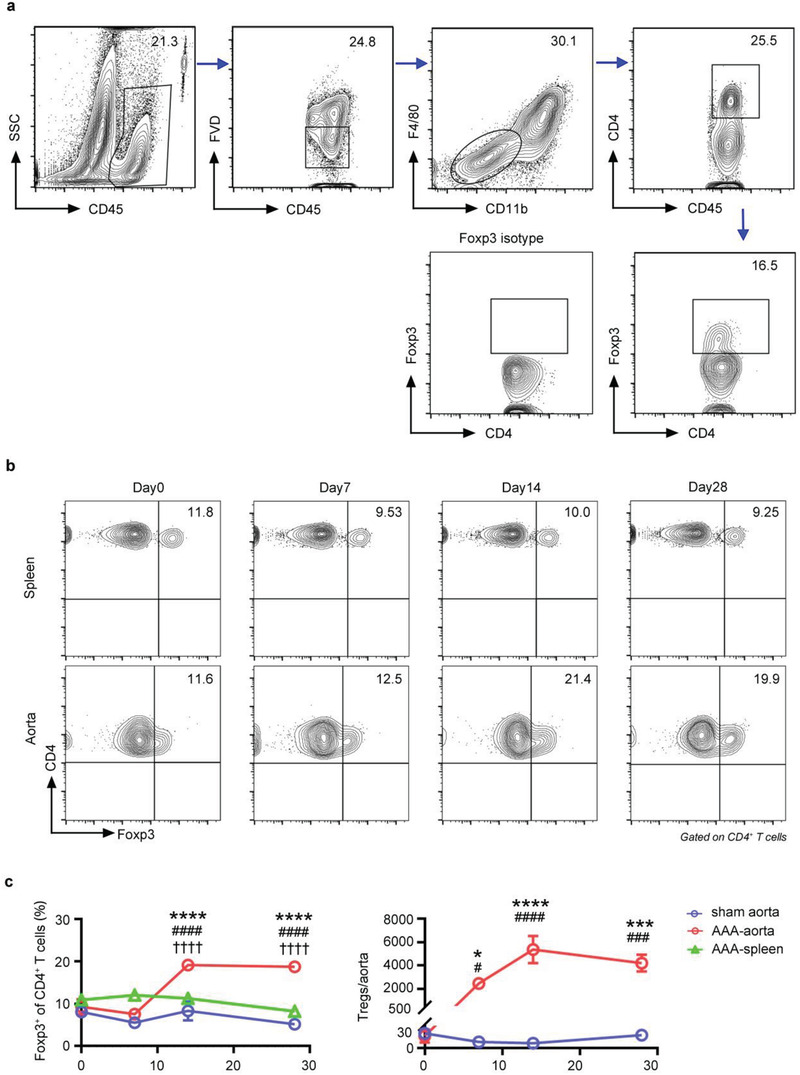
Treg accumulation in the aorta of AAA. a) Gating strategy for Tregs in the aorta by flow cytometry. Lymphocytes were gated out by CD45^+^, live cells were gated out by FVD^–^, CD45^+^FVD^–^F4/80^–^CD11b^–^ cells were gated out for CD4 and Foxp3 analysis. The number inside of gate indicates cell events. b) Flow cytometry analysis of spleen and aorta Tregs in AAA mice at different time points (day 0, 7, 14, 28). c) Percentages and numbers of Foxp3^+^ Tregs in the spleen and aorta of AAA mice at different time points (pregated for CD4^+^ T cells) (*n* = 4–6 per group), two‐way ANOVA, *p<0.05, ***p<0.001, ****p<0.0001 versus 0‐day post‐AAA; #*p*<0.05, ###*p*<0.001, ####*p*< 0.0001 versus sham‐operated aorta at each time point; ^††††^ *p*<0.0001 versus spleen at each time point. Data are presented as mean ± S.E.M.

It has been previously shown that Tregs play a protective role in angiotensin II‐induced AAA,^[^ ^16^, ^17^ ^]^ thus, determining Treg numbers in the aortas of mice is very important. However, only a few papers have tried to demonstrate the presence of Tregs in this tissue. We performed PPE (porcine pancreatic elastase)‐induced AAA and analyzed the dynamic change in Tregs in the abdominal aorta by flow cytometry^[^ ^18^ ^]^(**Figure** **1** **a**). The proportion of Tregs gradually expanded in the aorta during the process of AAA, reaching a peak level at 14 days postsurgery, and remained elevated at 28 days (Figure 1b,c). In contrast, the proportion of Tregs in the spleens showed little variability over the time course (Figure 1b,c). The number of Tregs began to rise 7 days after surgery, peaked at 14 days, and remained elevated at 28 days (Figure 1c). Moreover, the number of aortic CD4^+^ T cells and inflammatory cells both peaked at day 7 after AAA and decreased afterward (Figure S1a, Supporting Information), implying that the local inflammatory response was controlled, perhaps due to Tregs.

Having uncovered an increased population of Tregs infiltrating the aorta during AAA progression, we asked whether these cells played a role in AAA repair. We performed loss‐ and gain‐of‐function studies to evaluate the role of Tregs in PPE‐induced AAA. Tregs were selectively removed by utilizing DEREG mice in which the diphtheria toxin receptor is expressed specifically on Tregs. Ablation of Tregs markedly increased the aortic diameter (Figure S1b, Supporting Information) and TUNEL‐positive SMCs (Figure S1c, Supporting Information). In contrast, expansion of Tregs via IL‐2 complex treatment resulted in a smaller aortic aneurysm diameter (Figure S1d, Supporting Information) and fewer TUNEL‐positive SMCs (Figure S1e, Supporting Information). These results indicate that Tregs have an important protective role in PPE‐induced AAA.

### 2.2 The Transcriptomic Profile of Aorta Tregs Reveals a Unique Phenotype

**Figure 2:**
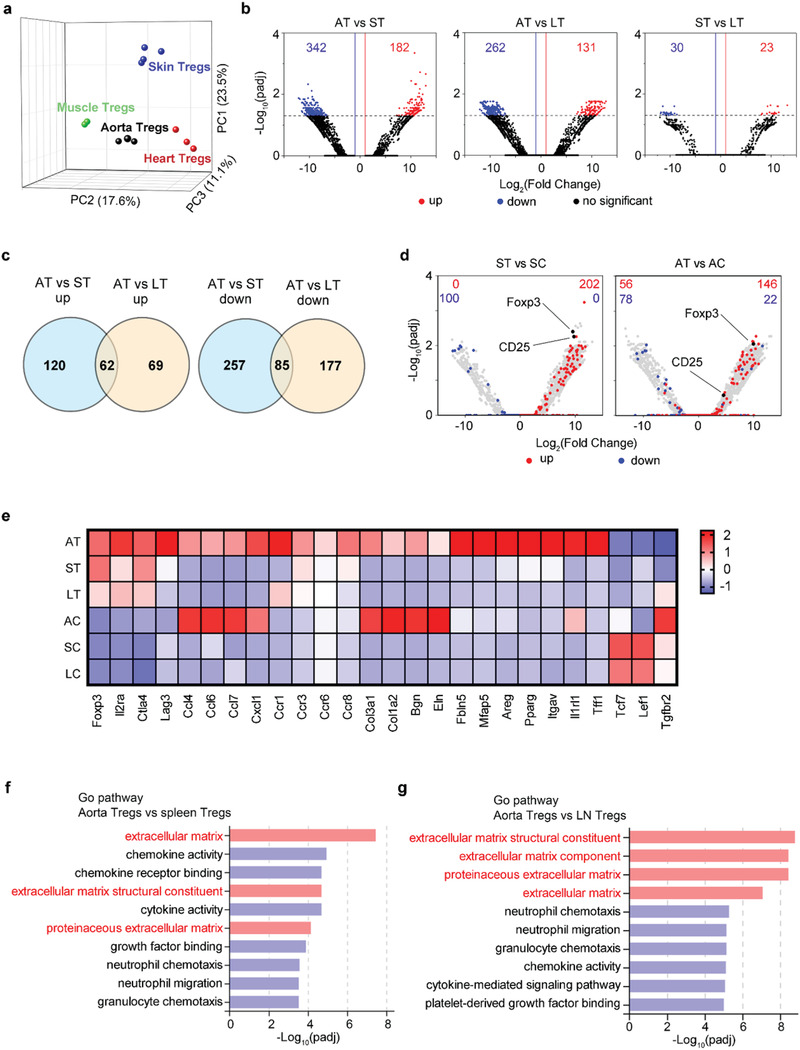
A unique phenotype of aorta Tregs. a) Principal component analysis of mRNA expression of different tissue Tregs from the post‐AAA aortas, injured muscle, post‐MI heart and hair‐regenerating skin. Each symbol represents one sort of pooled Tregs (muscle Tregs: GSE76733, heart Tregs: GSE155483 and skin Tregs: GSE76138). b) RNA sequencing was performed on Tregs isolated from the aorta, spleen, and LN of AAA mice. Volcano plots of normalized expression values. Numbers indicate the number of genes whose expression differed by more than 2‐fold. c) Venn diagram depicting the overlap of differentially expressed genes between aorta Tregs and spleen Tregs and between aorta Tregs and LN Tregs. d) Volcano plots comparing the gene expression of Tregs versus Tconvs. Treg signature genes are highlighted in red (induced) or blue (repressed). Numbers represent the number of signature genes expressed by each population. e) Heat map of the RNA‐seq analysis of Tregs and Tconvs from the aorta, spleen, and LN. Averaged from 3 experiments. f) Image shows the top 10 GO pathways determined by comparison of RNA sequencing data from aorta Tregs and spleen Tregs. g) Image shows the top 10 GO pathways determined by comparison of RNA sequencing data from aorta Tregs and lymphoid node Tregs. AT: aorta Tregs; ST: spleen Tregs; LT: lymphoid Tregs; AC: aorta Tconvs; SC: spleen Tconvs; LC: lymphoid Tconvs.

Given the special role of Tregs in AAA, we performed bulk RNA‐seq on Tregs to highlight the tissue specificity phenotype of aorta Tregs compared with Tregs in the spleen, lymph nodes (LNs) and other organs.^[^ ^8^, ^9^, ^12^ ^]^ According to principal component analysis, aorta Tregs from AAA mice differed significantly from other types of tissue Tregs, such as muscle,^[^ ^19^ ^]^ heart,^[^ ^14^ ^]^ and skin Tregs^[^ ^12^ ^]^(**Figure** **2** **a**). Volcano plots demonstrated that the transcriptome of aorta Tregs differed from that of their spleen or LN counterparts more than the latter two (Figure 2b). The differentially expressed genes between aorta Tregs and spleen Tregs and those between aorta Tregs and LN Tregs showed some overlap, which included 62 upregulated and 85 downregulated genes (Figure 2c). Although exhibiting a distinct gene expression profile, aorta Tregs were still clearly “Tregs”, showing the anticipated expression of 74% of the canonical Treg signature,^[^ ^20^ ^]^ in particular, characteristic transcripts such as those encoding Foxp3 and CD25 (Figure 2d).

Next, we explored the differences in the heat map of gene expression. Aorta Tregs showed high expression levels of several Treg signature genes, such as *Foxp3, Il2ra, Ctla4*, and *Lag3*. Chemokine ligand/receptor genes (*Ccl4*, *Ccl6*, *Ccl7*, *Cxcl1, Ccr1, Ccr3, Ccr6*, and *Ccr8*), extracellular matrix‐related genes (*Col3a1*, *Col1a2*, *Bgn*, *Eln, Fbln5*, and *Mfap5*) and tissue Treg signature genes (*Areg, Pparg, Itgav*, and *Il1rl1*) were highly expressed in aorta Tregs compared with their lymphoid organ counterparts. In contrast, some genes (*Tcf7, Lef1*, and *Tgfbr2*) with low expression in other tissue Tregs^[^ ^9^, ^13^ ^]^ were also poorly expressed in aorta Tregs (Figure 2e). Notably, we found that aorta Tregs specifically expressed high levels of Tff1, suggesting a possible role of Treg derived Tff1 in AAA (Figure 2e). We performed Gene Ontology (GO) pathway analysis and found that aorta Tregs no longer only played an immunomodulatory role but that their roles in AAA became related to regulating the extracellular matrix (Figure 2f,g). This finding suggested that aorta Tregs are a new group of tissue Tregs that differ from traditional lymphoid Tregs and perform specific functions associated with the tissue microenvironment.

### 2.3 Aorta Tregs are Clonally Expanded and Display a Unique TCR Repertoire

**Figure 3:**
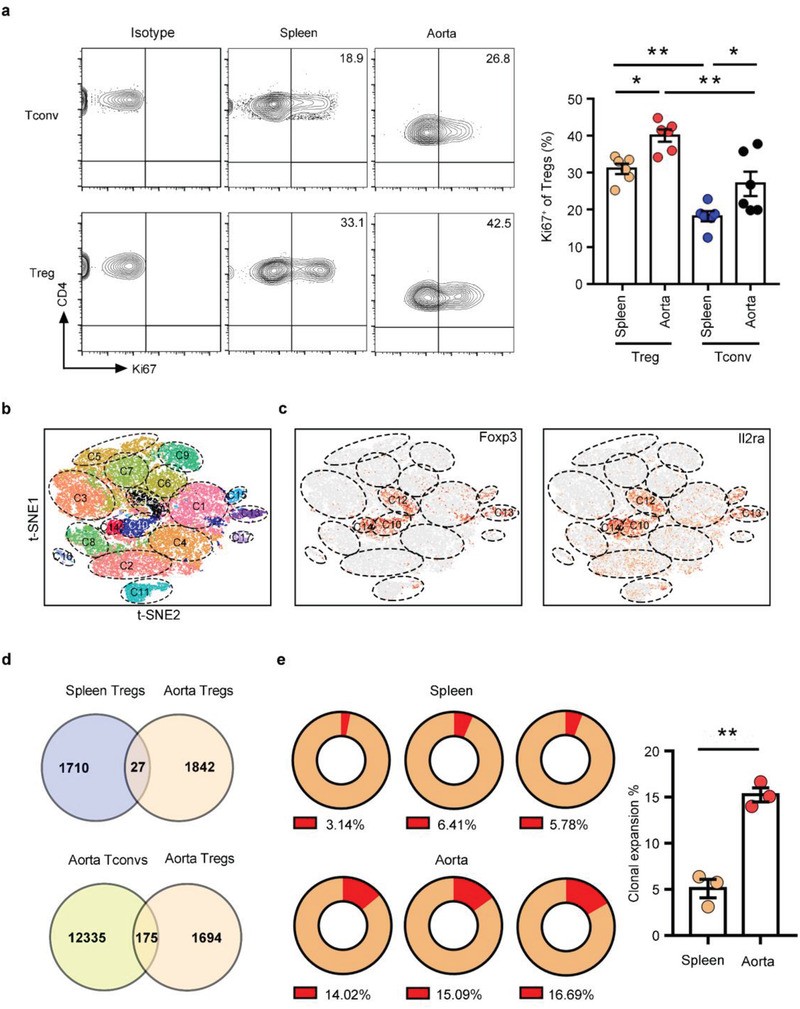
Aorta Tregs with a unique TCR repertoire are clonally expanded. a) Flow cytometry analysis of Ki67 expression in splenic and aortic Tregs/Tconvs from AAA mice. Representative FACS images are on the left, and quantification of the percentages is on the right (*n* = 6 per group), one‐way ANOVA, *p<0.05, **p<0.01. b) The t‐SNE projection showing the formation of 17 clusters. Each dot corresponds to a single cell, colored according to cell cluster. c) t‐SNE plot of the expression levels of selected genes (*Foxp3* and *Il2ra)* in different clusters. d) Venn diagram depicting the overlap of different TCRs between spleen Tregs and aorta Tregs and between aorta Tregs and aorta Tconvs. e) Percentages of clonal expansion in spleen Tregs and aorta Tregs. Repeated TCR clonotype proportion are presented in red in the pie chart and numbers in the bottom of the pie charts represent the proportion of the repeated cells in all cells analyzed. Aggregated percentages of clonal expansion in the spleen and aorta are shown on the right (*n* = 3 per group), unpaired 2‐tailed t test, **p<0.01. Data are presented as mean ± S.E.M.

The rapid accumulation of Tregs at the aorta could reflect their influx, proliferation, or some combination of the two. We assessed the proliferative state of Tregs in aortic aneurysm by flow cytometry. Compared with spleen Tregs, a much higher fraction of aorta Tregs proliferated, as shown by Ki67 staining (**Figure** **3** **a**). Aortic conventional T cells (Tconvs, defined as CD4^+^Foxp3^–^cells) in aortic aneurysm also exhibited an increased amount of Ki67 expression compared with the spleen Tconvs, however the fractions of Ki67^+^ within aorta Tconvs was significantly lower than that of the aorta Tregs, indicating that Tregs in AAA exhibited a proliferative phenotype (Figure 3a).

The T cell receptor (TCR) repertoire represents a parameter for assessing the degree of clonality of T cells. To clarify the clonality of aorta Tregs in AAA, we performed single‐cell RNA‐seq (scRNA‐seq) analysis, combined with TCR sequencing, on CD4^+^ cells from the aorta and corresponding spleen of AAA mice. Unsupervised t‐distributed stochastic neighbor embedding (t‐SNE) clustering of these 41341 single cells identified 17 statistically distinct populations (Figure 3b) and all expressed *Cd4* (Figure S2a, Supporting Information). We identified four Treg clusters (C10, C12, C13, and C14) according to the Treg‐specific transcripts (*Il2ra* and *Foxp3*) between aorta and spleen (Figure 3c). According to the tissue distribution (Figure S2b, Supporting Information), we analyzed the TCR repertoire of Tregs in the spleen and aorta. Similar to other tissue Tregs, aorta Tregs had a unique TCR phenotype, and only a few TCRs of aorta Tregs were the same as those of spleen Tregs or aorta Tconvs (Figure 3d). We found clonal expansion^[^ ^9^, ^21^ ^]^ of spleen Tregs and aorta Tregs, and the ratio of aorta Treg clonal expansion was significantly higher than that of spleen (Figure 3e), as expected from the results of Ki67 staining. The Gini inequality coefficient was calculated as an indicator of repertoire evenness and clonal dominance and the aorta Tregs displayed higher Gini scores than spleen Tregs (Figure S2c, Supporting Information). These results revealed that Treg responses were marked by clonal expansion in AAA. In addition, we discovered the same TCR in different mouse samples (Figure S2d, Supporting Information), suggesting that this TCR was probably directed to a specific antigen in the aorta, which was artery‐specific.

### 2.4 Increased Tregs During AAA Progression are Mainly Derived from Peripheral Recruitment

**Figure 4:**
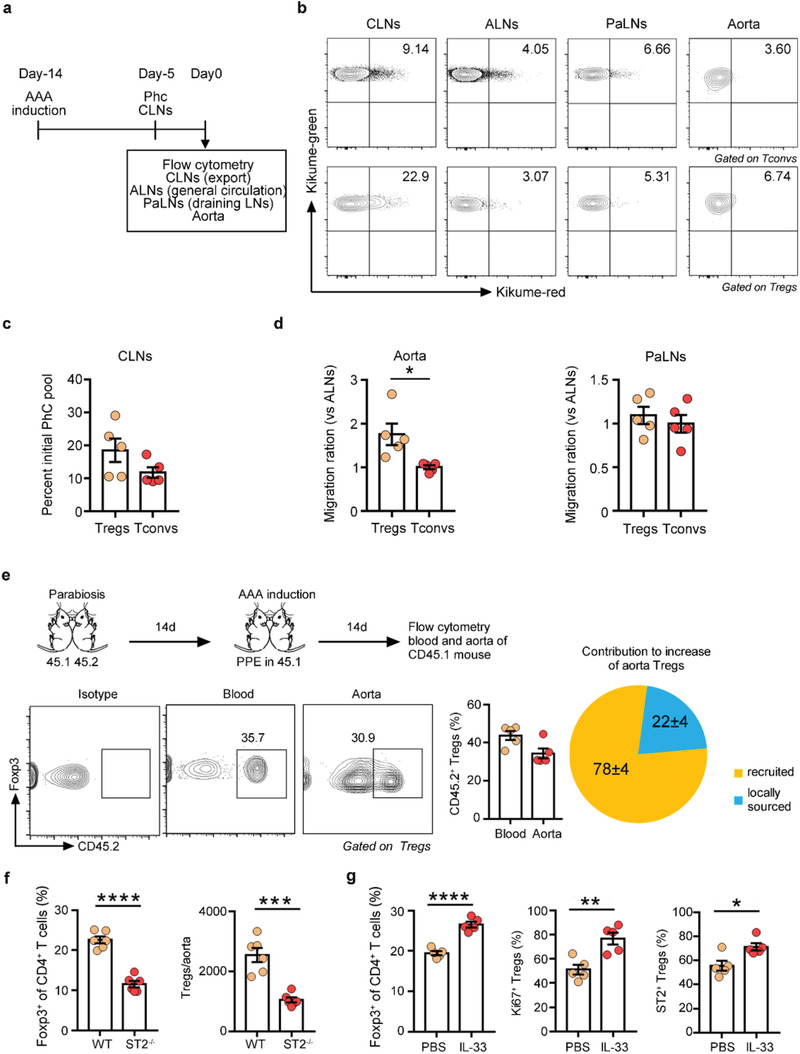
Increasing numbers of Tregs through the AAA process are mainly derived from peripheral recruitment. a) Schematic diagram of the photoconversion experiments. AAA was induced in the KikGR mice. On the fifth day before examination, the CLNs were exposed to violet light noninvasively. Tregs or Tconvs from the indicated tissues were examined by flow cytometry to track Kikume red^+^ photoconverted (PhC) cells. b) Representative FACS images of Kikume red^+^ cells in the CLNs, ALNs, PaLNs and aorta pregated for CD4^+^CD25^–^ Tconvs (top) or CD4^+^CD25^+^ Tregs (bottom). c) Exodus from the PhC CLN pool. d) Migration ratio of aorta Tregs and Tconvs or PaLN Tregs and Tconvs (*n* = 5 per group). The “‘migration ratio”’ is the fraction of Kikume red^+^ cells in a designated organ normalized to the fraction in the nondraining LN (ALNs), which provides a measure of systemic circulation, unpaired 2‐tailed t test, *p<0.05. e) B6 CD45.1 and B6 CD45.2 mice were conjoined for 2 weeks. Then, we conducted PPE‐induced surgery in the CD45.1 parabiont and analyzed the chimerism of CD45.2^+^ Tregs in the blood and aorta 2 weeks later. The experimental procedure and timeline of surgery are shown on top. Representative FACS images and quantification are on the bottom (*n* = 5 per group). The results are a combination of two independent experiments. f) Flow cytometry analysis of aortic Tregs in WT and ST2^–/–^ mice 14 days after AAA surgery (*n* = 6 per group), unpaired 2‐tailed *t* test, ***p<0.001, ****p<0.0001. g) Flow cytometry analysis of aortic Tregs and their ST2/Ki67 expression in AAA WT mice treated with PBS or IL‐33 (*n* = 5 per group), unpaired 2‐tailed t test, *p<0.05, **p<0.01, ****p<0.0001. Data are presented as mean ± S.E.M.

Having identified the active proliferation of aorta Tregs, we explored other provenances of Tregs in AAA. On the one hand, it was unclear whether and to what extent tissue Tregs communicated with the pool of circulating T cells. First, we used FTY720, an S1P1 receptor agonist, to prevent T cell recirculation from lymphoid organs to peripheral sites,^[^ ^19^ ^]^ and the proportion and number of aorta Tregs were significantly reduced after the administration of FTY720 (Figure S3a, Supporting Information). Next, we employed KikGR/B6‐ROSA transgenic mice to track the migration of Tregs and Tconvs in AAA. These mice ubiquitously express a photoconvertible fluorescent reporter that can be irreversibly converted from green to red innocuously and stably for at least 2 weeks upon exposure to violet light.^[^ ^22^ ^]^ Noninvasively photoconverting cells in the cervical lymph node (CLN) of KikGR mice by exposure to violet light, Kikume‐red^+^ Tregs and Tconvs were tracked from the CLN to the axillary LN (ALN, general circulation), the para‐abdominal aortic lymph node (PaLN, abdominal aorta‐draining), and the aorta (**Figure** **4** **a**). We detected a group of Kikume‐red^+^ Tregs and Tconvs in the ALNs, PaLNs and aortic aneurysm (Figure 4b,c and Figure S3b, Supporting Information). When the PaLN and AAA fractions were normalized to the ALN values to correct for general circulation patterns, it became clear that the major difference between Tregs and Tconvs was due to the migration of Tregs to the injured aorta (Figure 4d). Finally, to determine the extent to which peripheral circulating Tregs contributed to the aorta Treg population, we performed parabiosis experiments. B6 CD45.1 and B6 CD45.2 mice were conjoined for two weeks. When the parabionts established shared circulation, we conducted PPE‐induced surgery in the CD45.1 parabiont (Figure 4e). After two weeks, we collected peripheral blood and aortas from CD45.1 mice for flow cytometry analysis and compared the chimaerism of CD45.2^+^ Tregs in the blood and aorta.^[^ ^23^ ^]^ In the aorta of AAA mice, the proportion of recruited Tregs to the Treg population in PPE parabionts was close to the proportion in the peripheral blood. Based on these data, we estimated that the recruited Tregs contributed approximately 78% of the expanded Treg population to the aorta during AAA progression (Figure 4e).

On the other hand, it was unknown whether Tregs in AAA were thymus‐derived Treg (tTreg) cells that develop in the thymus or peripherally induced Treg (pTreg) cells that were derived from converted Tconvs in peripheral tissues. Tregs in AAA had a Helios^hi^Neuropilin (Nrp)‐1^hi^ phenotype (Figure S3c, Supporting Information), indicating a thymic source.^[^ ^24^, ^25^ ^]^ Additionally, aorta Tregs and Tconvs shared some TCRs (Figure 3d), indicating a possible conversion of Tconvs to Tregs. Hence, we transferred CD45.2^+^ Tconvs (CD4^+^CD45.2^+^Foxp3^–^) into CD45.1 mice and observed a small population of donor‐derived CD45.2^+^ Tregs (CD4^+^CD45.2^+^Foxp3^+^) in aortic aneurysms, spleens and lymph nodes, and the frequency of CD45.2^+^ Tregs in aortic aneurysms was higher than that in lymphoid organs (Figure S3d, Supporting Information), verifying the conversion of Tconvs to Tregs in AAA.

### 2.5 The IL‐33/ST2 Axis is Involved in the Maintenance of Aorta Tregs

Next, we focused on the molecular mechanism of sustained aorta Tregs. Several studies have highlighted the role of IL‐33 and its receptor, ST2, in the regulation of tissue Tregs.^[^ ^19^, ^21^, ^26^ ^]^ Our previous study found that the expression of IL‐33 and ST2 was increased in aneurysms, and IL‐33 was mainly derived from fibroblasts.^[^ ^26^ ^]^ Here, we further explored the expression of ST2 on aorta Tregs in aneurysms and observed the roles of the IL‐33/ST2 axis on aorta Tregs by enhancing or blocking the IL‐33/ST2 signaling pathway.

Aorta Tregs specifically expressed high levels of ST2 (Figure S4a, Supporting Information). In ST2‐deficient mice, Tregs in the injured aorta were decreased (Figure 4f), while Tregs in the spleen remained unchanged (Figure S4b, Supporting Information). In contrast, aorta Tregs showed increased proliferation and elevated expression of ST2 in response to exogenous administration of IL‐33 in AAA WT mice (Figure 4g). In conclusion, these data suggested that the IL‐33/ST2 axis was involved in the maintenance of aorta Tregs.

### 2.6 A Similar Treg Population is Expanded in a CaPO_4_‐Induced AAA Model

**Figure 5:**
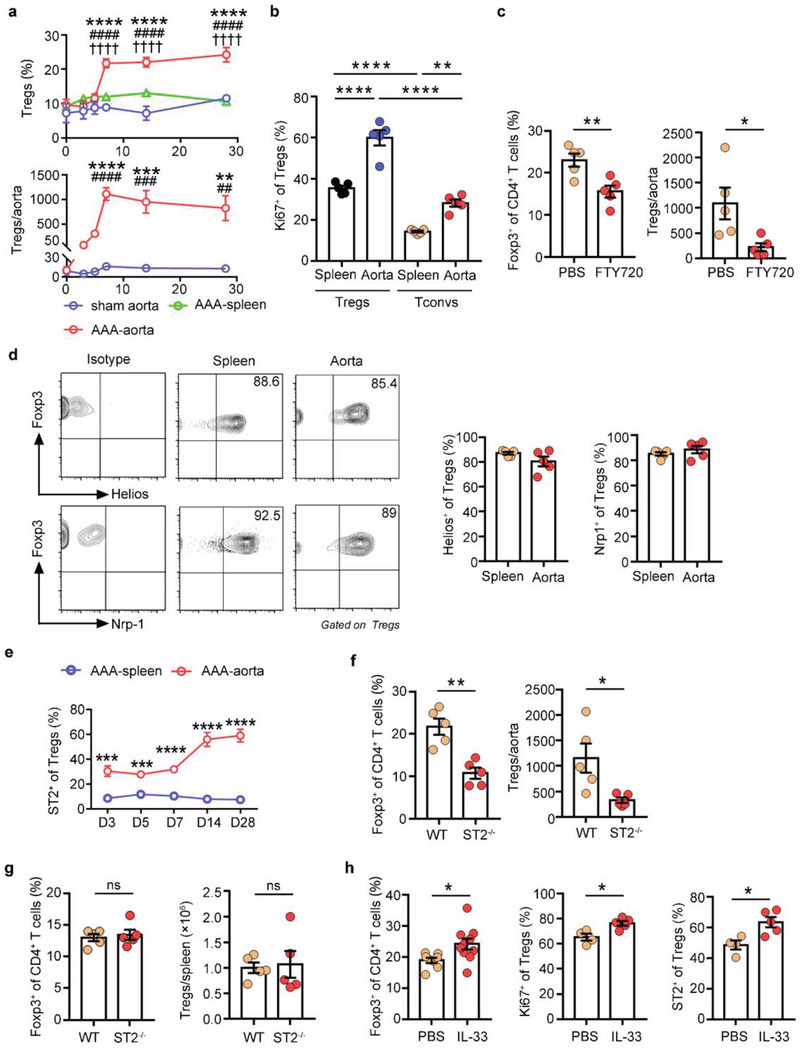
A similar Treg population is expanded in the CaPO_4_‐induced AAA model. a) Percentages and numbers of Foxp3^+^ Tregs in the spleen and aorta in AAA mice at different time points (days 0, 3, 5, 7, 14, and 28; *n* = 3–5 per group). two‐way ANOVA, **p<0.01, ***p<0.001, ****p<0.0001 versus 0‐day post‐AAA; ##*p*< 0.01, ###*p*< 0.001, ####p< 0.0001 versus sham‐operated aorta at each time point; ^††††^ *p*< 0.0001 versus spleen at each time point. b) Flow cytometry analysis of Ki67 expression in spleen and aorta Tregs/Tconvs of AAA mice (*n* = 5 per group), one‐way ANOVA, **p<0.01, ****p<0.0001. c) Flow cytometry analysis of aorta Tregs in CaPO_4_‐induced AAA mice treated with PBS or FTY720 (*n* = 5 per group), unpaired 2‐tailed *t* test, *p<0.05, **p<0.01. d) Flow cytometry analysis of Helios and Nrp‐1 expression in spleen and aorta Tregs of AAA mice. Representative FACS images are on the left, and quantification of the percentages is on the right (*n* = 5 per group). e) Quantification of the percentages of ST2 expression on spleen and aorta Tregs in AAA mice at different time points by flow cytometry (*n* = 5 per group), unpaired 2‐tailed *t* test, ***p<0.001, ****p<0.0001 versus spleen at each time point. f) Percentages and numbers of aorta Tregs in WT and ST2^–/–^ mice after 7 days of AAA induction (*n* = 5 per group), unpaired 2‐tailed *t* test, *p<0.05, **p<0.01. g) Percentages and numbers of spleen Tregs in WT and ST2^–/–^ mice after 7 days of AAA induction (*n* = 5 per group), unpaired 2‐tailed *t* test, ns: not significant. h) Percentages of aorta Tregs (pregated for CD4^+^ cells) and ST2/Ki67 expression (pregated for CD4^+^Foxp3^+^ cells) in AAA WT mice treated with PBS or IL‐33 (*n* = 4–10 per group). The results are a combination of two independent experiments, unpaired 2‐tailed *t* test, *p<0.05. Data are presented as mean ± S.E.M.

To confirm whether aorta Tregs broadly existed, we produced CaPO_4_‐induced AAA to assess aorta Tregs. Consistently, in this model, we found that a similar Treg population accumulated in aortic aneurysms and showed a high proliferation state (**Figure** **5**a,b). We then tested whether Tregs in the CaPO_4_‐induced model also had the same source as those in the PPE model. Administration of FTY720 blocked the increase in Tregs in AAA (Figure 5c) and this observation demonstrated that the circulating Treg pool contributed substantially to the aorta Treg population. Additionally, we examined the expression of Helios and Nrp‐1 on aorta Tregs in this model and found that these Tregs expressed these two molecules at high levels (Figure 5d), suggesting a thymic origin.

We next tested whether the IL‐33/ST2 axis was also involved in the maintenance of aorta Tregs in CaPO_4_‐induced AAA. Compared with spleen Tregs, aorta Tregs highly expressed ST2 (Figure 5e). In ST2‐deficient mice, aorta Tregs were significantly reduced (Figure 5f), while the spleen Tregs did not change (Figure 5g). Recombinant IL‐33 increased the expansion and proliferation of aorta Tregs and enhanced ST2 expression in AAA WT mice (Figure 5h). These results suggested that IL‐33/ST2 axis was essential for maintaining aorta Tregs in CaPO_4_‐induced AAA.

Taken together, these observations suggest that aorta Tregs exist as a unique population in aortic aneurysms, independent of AAA models.

### 2.7 Aorta Tregs Preferentially Expressed Tff1 and Inhibited SMC Apoptosis to Attenuate AAA

**Figure 6:**
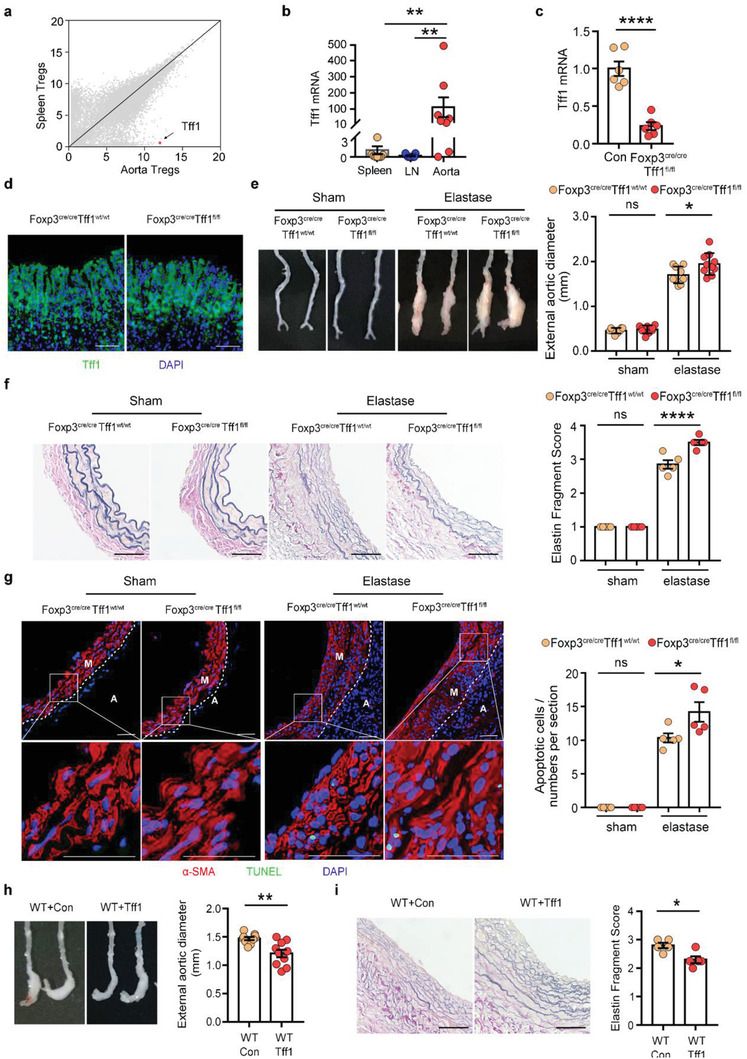
Tff1 produced by Tregs regulates SMC apoptosis in AAA. a) Scatter plots comparing gene expression quantified by RNA sequencing of aorta Tregs versus spleen Tregs. The Tff1 gene is highlighted in red. b) RT‐PCR assay of Tregs from spleen, LN, and aorta in AAA mice 14 days after AAA induction by SMART‐Seq (*n* = 8–9 per group), Kruskal‐Wallis test, **p<0.01. (c) RT‐PCR analysis of Tff1 mRNA levels in Tregs of Foxp3^cre/cre^Tff1^wt/wt^ and Foxp3^cre/cre^Tff1^fl/fl^ mice (*n* = 6 per group), unpaired 2‐tailed *t* test, ****p<0.0001. d) Immunofluorescent image of the expression of Tff1 in the stomach from the 2 groups of mice. Tff1 (green); DAPI (blue), scale bar: 50 µm. e) Representative photographs of aortic fragments from the four groups: Foxp3^cre/cre^Tff1^wt/wt^/sham (*n* = 8), Foxp3^cre/cre^Tff1^fl/fl^ /sham (*n* = 9), Foxp3^cre/cre^Tff1^wt/wt^ /elastase (*n* = 9), Foxp3^cre/cre^Tff1^fl/fl^ /elastase (*n* = 10) and quantification of the maximal external diameter of the infrarenal aortas, one‐way ANOVA, *p<0.05. f) Representative images of elastic Verhoeff‐Van Gieson (EVG) staining of aortic tissue from the 4 groups of mice and the assessment of medial elastica fragmentation (*n* = 5 per group), one‐way ANOVA, ****p<0.0001, scale bar: 50 µm. g) Representative immunofluorescent image of SMC apoptosis from the 4 groups of mice. Left: *α*‐SMA (red) is costained with TUNEL (green). Right: Quantification of the numbers of apoptotic SMCs (*n* = 5 per group), one‐way ANOVA, *p<0.05, ns: not significant, scale bar: 50 µm. M: media, A: adventitia. h) Representative photographs of aortic fragments from the 2 groups of mice (left) and the quantification of the maximal external diameter of the infrarenal aortas (right) (*n* = 10 per group), unpaired 2‐tailed *t* test, **p<0.01. i) Representative images of EVG staining of aortic tissue from the 2 groups of mice (left) and the assessment of medial elastica fragmentation (right) (*n* = 5 per group), unpaired 2‐tailed *t* test, *p<0.05, scale bar: 50 µm. Data are presented as mean ± S.E.M.

Tregs have been reported to play a role in protection against AAA formation by suppressing inflammation. In addition to regulating immune responses, tissue Tregs also have the capacity to directly influence surrounding parenchymal cells. To determine the tissue‐specific roles of aorta Tregs in AAA, we analyzed the transcriptomic data of aorta Tregs. Interestingly, Tff1, a key peptide for gastrointestinal protection and repair,^[^ ^27^, ^28^ ^]^ was among the highest differentially expressed genes produced by aorta Tregs versus lymphoid organ Tregs (**Figure** **6** **a** and Figure S5a,b, Supporting Information). Previous studies and examination of the BioGPS database (http://biogps.org) (Figure S5c, Supporting Information) indicated that *Tff1* was mainly expressed by gastric mucosal epithelial cells. However, we found that *Tff1* was mainly expressed by CD45^+^ cells in AAA tissue at day 14 (Figure S5d, Supporting Information). In addition, after depleting Tregs by using an anti‐CD25 antibody (Figure S5e, Supporting Information) and DEREG mice (Figure S5f, Supporting Information), we found that the expression of *Tff1* was significantly decreased in AAA tissue at day 14. To confirm the expression of *Tff1* on aorta Tregs, we performed RT‐PCR assays, and the results revealed that aorta Tregs expressed significantly higher levels of *Tff1* than spleen Tregs and LN Tregs in AAA mice at day 14 (Figure 6b). Next, we analyzed the single‐cell sequencing data which was published in GEO database (GSE152583),^[^ ^29^ ^]^ and the results (Figure S5g, Supporting Information) suggested that almost all cells expressing Tff1 expressed Foxp3, while other immune cells like CD4^+^ T cells and macrophages hardly expressed Tff1, further confirming our results that Tregs were the main source of Tff1 in AAA. In addition, we detected the expression of Tff1 on Tregs by using immunofluorescence, and confirmed that Tff1 could be produced by Tregs in aortic aneurysm (Figure S5h, Supporting Information). Therefore, specific secretion of Tff1 by aorta Tregs may indicate a tissue‐specific function.

Having elucidated the expression of Tff1 in aorta Tregs, we next sought to functionally determine whether Tff1 plays a role in Treg‐mediated vascular repair in AAA. Previous studies have shown that Tff1 can suppress cell apoptosis^[^ ^30^, ^31^ ^]^ and SMC apoptosis is one of the most important pathogenic mechanisms of AAA. Thus, we speculated that Tff1 produced by Tregs played an anti‐apoptotic role in AAA. To clarify the role of Treg‐derived Tff1 in AAA and determine whether it affected SMC apoptosis, mice expressing a Tff1 conditional allele (Tff1^flox/flox^) were crossed with Foxp3‐cre mice to specifically ablate Tff1 expression in Tregs (Figure 6c), while the expression of Tff1 in the stomach was not affected in these mice (Figure 6d). Then Foxp3^cre/cre^Tff1^flox/flox^ mice were subjected to PPE‐induced AAA. After conditionally knocking out Tff1 in Tregs, the protective effect of aorta Tregs was attenuated, as indicated by increased aneurysm diameter (Figure 6e), extensive degradation of elastin in the media of the vessel wall (Figure 6f) and increased apoptosis of SMCs (Figure 6g), and there was no difference in the sham operated group (Figure 6e–g).

Since the Foxp3‐cre mice may also express Cre in other inflammatory cells, we performed Treg adoptive transfer assay to provide further supports of the current data. We obtained the spleen Tregs from WT mice and Tff1^KO^ mice and adoptively transferred them to DEREG mice. After 14 days of AAA induction, we found that the recipient mice transferred with Tff1^KO^ Tregs had a larger aneurysm diameter than mice transferred with WT Tregs (Figure S6a, Supporting Information). Degradation of elastin (Figure S6b, Supporting Information) and apoptosis of SMCs (Figure S6c, Supporting Information) were also reduced in the recipient mice transferred with Tff1^KO^ Tregs, further suggesting that Tff1 derived from Tregs has an important role in AAA protection.

Then, we evaluated the therapeutic role of Tff1 in AAA by adenovirus overexpression. Intravenous injection of the Tff1‐adenovirus effectively increased the expression of Tff1 in the aorta (Figure S6d, Supporting Information). As expected, Tff1 treatment played a protective role in AAA (Figure 6h,i). After overexpression of Tff1 in WT mice, the apoptosis of SMCs was significantly reduced compared to that of the control group (Figure S6e, Supporting Information). These results suggested that Tff1 may have potential clinical translational value.

### 2.8 Tff1 Inhibited SMC Apoptosis via Activation of the ERK1/2 Signaling Pathway

**Figure 7:**
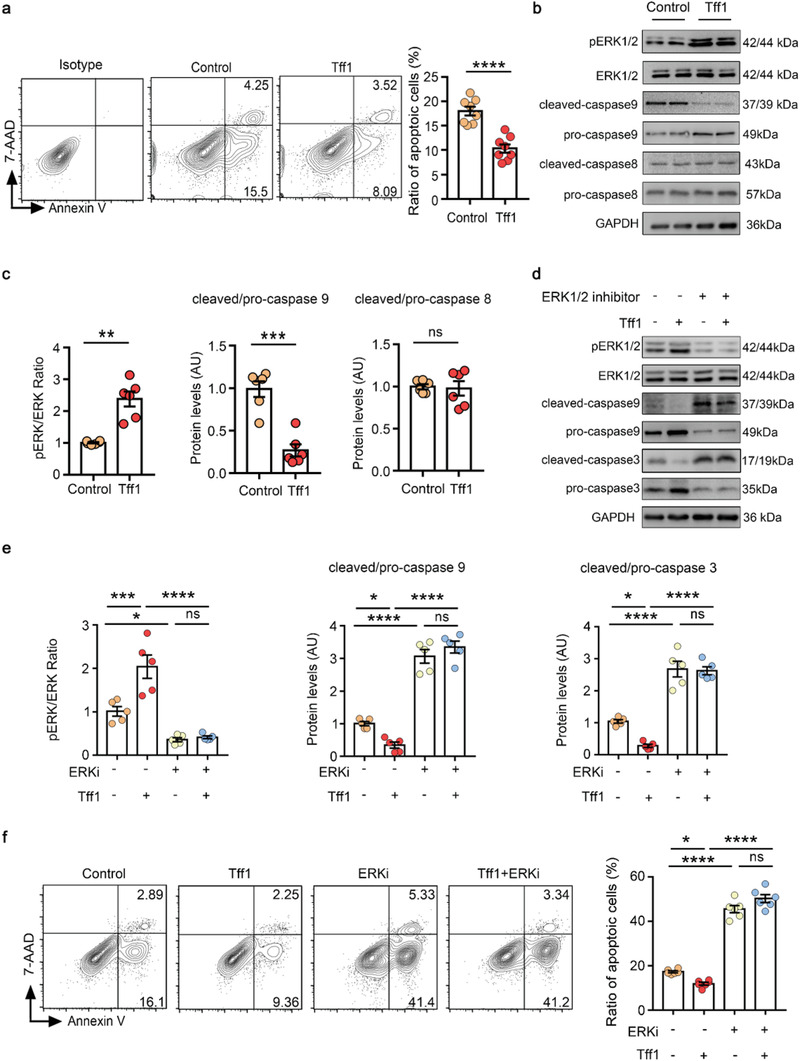
Tff1 inhibited SMC apoptosis via the activation of ERK1/2 pathway. a) Tff1 (500 ng mL^−1^) was used to treat the primary mouse VSMCs and TNF‐*α* (100 ng mL^−1^) was used to induce apoptosis, and apoptosis was detected by flow cytometry 24 h later. Representative FACS images are on the left, and quantification of the percentages is on the right (*n* = 8 per group), unpaired 2‐tailed *t* test, ****p<0.0001. b,c) Representative immunoblots (b) and quantification (c) for phosphorylated, total ERK, pro/cleaved caspase‐9 and pro/cleaved caspase‐8 (*n* = 6 per group), unpaired 2‐tailed *t* test, **p<0.01, ***p<0.001, ns: not significant. d,e) Representative immunoblots (d) and quantification (e) for phosphorylated and total ERK, pro/cleaved caspase‐9 and pro/cleaved caspase‐3 (*n* = 5 per group), one‐way ANOVA, *p<0.05, ***p<0.001, ****p<0.0001. f) Tff1 (500 ng mL^−1^) and ERK1/2 inhibitor (ERKi: 10 µmol mL^−1^) were used to treat the VSMCs and TNF‐*α* (100 ng mL^−1^) was used to induce apoptosis, apoptosis was detected by flow cytometry 24 h later. Representative FACS images are on the left, and quantification of the percentages is on the right (*n* = 6 per group), one‐way ANOVA, *p<0.05, ****p<0.0001, ns: not significant. AU: arbitrary units. Data are presented as mean ± S.E.M.

Since our in vivo experiments showed that Tregs could inhibit SMC apoptosis, we next investigated whether Tff1 had a direct effect on primary mouse vascular smooth muscle cells (VSMCs) in vitro. The purity of the VSMC preparations was confirmed by immunostaining using an SMC marker anti‐mouse *α*‐SMA antibody (Figure S6f, Supporting Information). In cultured VSMCs, Tff1 attenuated the apoptosis of SMCs (**Figure** **7** **a**) in a concentration‐ and incubation time‐dependent manner (Figure S6g, Supporting Information). Extracellular signal‐regulated kinase (ERK) 1/2, AKT, and NF‐*κ*B pathways have been reported to exert an important role in SMC survival;^[^ ^32^, ^33^, ^34^, ^35^ ^]^ however, the loss of Tff1 can upregulate the activation of AKT and NF‐*κ*B pathways, and the expression of Tff1 is positively correlated with the activation of ERK1/2.^[^ ^36^, ^37^, ^38^ ^]^ Thus, we speculated that Tff1 inhibited VSMC apoptosis through the ERK1/2 signaling pathway. We performed western blotting and found that Tff1 significantly increased the protein levels of phosphorylated ERK1/2 (pERK1/2) in VSMCs compared with the control group (Figure 7b,c). Caspases are key mediators of apoptosis. Caspase‐8 mediates extrinsic apoptosis, and caspase‐9 initiates the intrinsic pathway of apoptosis. To test whether caspase‐8 and/or caspase‐9 are involved in the antiapoptotic effects of Tff1, western blotting was used to detect the changes in the levels of caspase‐8 and caspase‐9. Compared with the control group, the levels of cleaved/pro‐caspase‐9 in the Tff1 group were significantly decreased, whereas cleaved/pro‐caspase‐8 was not changed (Figure 7b,c).

To further confirm the involvement of ERK1/2 signaling in Tff1‐mediated VSMC survival, VSMCs were treated with or without an ERK1/2 inhibitor (SCH772984) in the presence of Tff1. Tff1 significantly increased the phosphorylation of ERK1/2 and reduced cleaved/pro‐caspase‐9 and cleaved/pro‐caspase‐3 ratios, while ERK1/2 inhibitor significantly reversed these changes (Figure 7d,e). Furthermore, we clarified that the ERK1/2 inhibitor significantly increased VSMC apoptosis and eliminated the protective effects of Tff1 by flow cytometry (Figure 7f). Thus, Tff1 inhibited intrinsic apoptosis of VSMCs through activation of the ERK1/2 signaling pathway.

## 3 Discussion and Conclusion

An emerging body of literature suggests that Tregs represent a highly complex and heterogeneous lineage of lymphocytes that possess specialized functions in the tissues in which they reside. Here, we reported a distinct group of mouse AAA lesion‐specific Tregs: they obtained unique transcriptomes and TCR repertoires, and exerted an essential role in maintaining vascular tissue homeostasis beyond their well‐established role in suppressing inflammation in AAA. Specifically, the ability of Tregs to preserve vascular structural and functional integrity during AAA occurs through the production of Tff1 to inhibit SMC apoptosis.

What are the features of aorta Tregs in nature? We provided deep insight into the tissue‐specific characteristics of aorta Tregs: i) How are aorta Tregs distinguished from their lymphoid organ counterparts? ii) What is the provenance and maintenance mechanism? iii) How do aorta Tregs exert their distinctive function? Collectively, we verified that aorta Tregs were in compliance with the general principals of tissue Tregs.

According to the criteria that set tissue Tregs apart from classical Tregs, their tissue‐specific transcriptome and TCR repertoire, aorta Tregs can be distinguished from their lymphoid organ counterparts. First, the transcriptome of aorta Tregs was distinct from that of lymphoid organ Tregs, and differential gene analysis revealed that aorta Tregs exhibited tissue‐specific functions and expressed molecules related to the aortic microenvironment, which are important components of the extracellular matrix and are mainly secreted and synthesized by parenchymal cells of the aortic wall to maintain tissue integrity. These transcriptomic characteristics implied that aorta Tregs not only exerted anti‐inflammatory effects to limit excessive inflammatory responses, but also maintained homeostasis by cooperating with parenchymal cells in the aortic microenvironment to accelerate tissue repair, which was not available in lymphoid organ Tregs. Second, aorta Tregs display a distinct repertoire of TCRs. Compared to cells in the corresponding lymphoid organs, aorta Tregs exhibited higher clonal expansion. The TCR sequence profile of aorta Tregs contrasted starkly with that of spleen Tregs and aorta Tconvs, for which there were only a few shared clone types, implying a tissue‐specific TCR:Ag recognition pattern for aorta Tregs.

The rapid accumulation of Tregs at the aortic aneurysm could reflect their influx, proliferation, or some combination of the two. Although we demonstrated that aorta Tregs were mainly derived from peripheral recruitment through various experiments, we did not negate the contribution of local expansion of aorta Tregs on the increased number of Tregs in the aortic aneurysm. Actually, we confirmed the local expansion of aorta Tregs using Ki67 and single cell TCR sequencing, suggesting that the expansion of Tregs in the aorta is also the cause of the increase of aorta Tregs. It has been reported that the recruitment of Tregs to parenchymal tissues almost certainly reflects their response to chemokine gradients. Compared to lymphoid Tregs, aorta Tregs strongly expressed *Ccr1*, *Ccr3*, *Ccr6* and *Ccr8*, which might imply a tissue‐specific chemotactic pattern in aorta Tregs.^[^ ^8^, ^9^, ^13^ ^]^ Moreover, there was a certain amount of conversion of aorta Tregs, indicating that the local microenvironment may be friendlier to Tregs, which not only increased the recruitment of Tregs but also promoted the conversion of Tconvs to Tregs to respond to tissue damage.

Some unknown antigenic molecules can stimulate local Tregs, boosting their activation and promoting their proliferation to cope with tissue damage. Given the previously documented fact that TCR abundance in VAT Tregs and muscle Tregs was significantly lower than that in lymphoid tissue,^[^ ^9^, ^21^ ^]^ it is easy to speculate that TCR antigen recognition is an important factor driving the accumulation of Tregs in AAA. Interestingly, we found shared TCRs in aorta Tregs from different samples, suggesting that aorta Tregs might respond to a specific antigen in situ. The heart‐specific antigen^[^ ^39^ ^]^ and atherosclerosis‐related antigen^[^ ^40^, ^41^, ^42^ ^]^ found in previous studies have important potential value for the diagnosis and treatment of disease. Therefore, revealing AAA‐related antigens may translate laboratory results into clinical approaches for the diagnosis or treatment of AAA.

In addition to controlling the inflammatory response, tissue Tregs could directly target parenchymal cells via specific factors to maintain tissue homeostasis. A previous study indicated that Tff1 was upregulated in various pathological conditions, including inflammation and cancer^[^ ^27^ ^]^ and was mainly produced by parenchymal cells such as epithelial cells. However, we found that *Tff1* was among the most differentially expressed genes produced by aorta Tregs versus lymphoid organ Tregs, and we confirmed that the expression of Tff1 was limited to aorta Tregs, while lymphoid Tregs did not express Tff1. As for what stimuli in AAA triggered Tff1 expression in aorta Tregs, antigens and inflammatory factors might induce the expression of molecules in tissue Tregs.^[^ ^11^, ^43^ ^]^ Based on the fact that Tff1 was only expressed on aorta Tregs and not in other tissue Tregs, we speculated that local antigen stimulation might play a more important role in inducing aorta Tregs to express Tff1. This needs to be further explored. Although Tff1 is widely considered a tumor suppressor in gastric carcinogenesis, it has not yet been investigated in AAA. Tff1 has been reported to suppress apoptosis, in rat IEC18 diploid intestinal cells, human HCT116 colon cancer cells and AGS gastric cancer cells.^[^ ^28^ ^]^ Our results highlighted that Tff1 produced by aorta Tregs inhibited SMC apoptosis to prevent the progression of AAA. Furthermore, in vitro experiments confirmed that Tff1 inhibited SMC apoptosis through the ERK1/2 pathway. An exogenous increase in Tff1 in WT mice exerted similar effects, indicating the clinical value of Tff1 as a nonsurgical therapeutic target in AAA. Our study suggested that Tregs have the capacity to directly perform a tissue repair function, at least in part through the production of Tff1.

There has been no breakthrough in the nonsurgical treatment of human AAA, which has prompted us to search for effective ways to limit arterial dilatation and rupture. Beyond suppressing the inflammatory response, our studies reveal potential tissue reparative functions of Tregs via Tff1. To date, the use of Tregs and their products in clinical treatment has not been satisfactory, possibly because of inadequate knowledge of tissue‐specific Tregs. Aorta Tregs play a specific role in preventing the progression of AAA. Thus, the induction of AAA‐specific Tregs may yield novel concepts or strategies for future medical treatment of aneurysms.

## 4 Experimental Section

### Mice

Male C57BL/6J and CD45.1 mice aged 8–12 weeks were purchased from the Model Animal Research Center of Nanjing University (Nanjing, China). Foxp3‐GFP mice were kindly provided by Dr. Rudensky AY (Howard Hughes Medical Institute, Immunology Program, and Ludwig Center, Memorial Sloan Kettering Cancer Center, New York, NY). ST2^–/–^ mice on a C57BL/6J background were obtained from Dr. Andrew McKenzie (Medical Research Council Laboratory of Molecular Biology, University of Cambridge, Cambridge, U.K.). KikGR/B6‐ROSA transgenic mice were purchased from RIKEN BioResource Research Center (Tokyo, Japan). Depletion of regulatory T cells (DEREG) and Foxp3^YFP‐cre^ mice were purchased from the Jackson Laboratory (Bar Harbor, ME). Tff1^flox/flox^ mice were established and Tff1^–/–^ mice were purchased by Cyagen (Suzhou, China). Treg‐specific Tff1 knockout mice were created by crossing Tff1^flox/flox^ mice with Foxp3^YFP‐cre^ mice. The incidence of AAA in male mice is higher than in female mice,^[^ ^44^, ^45^ ^]^ so only male mice were used throughout this study. All animal studies were approved by the Animal Care and Utilization Committee of Huazhong University of Science and Technology. Experiments were conducted in accordance with NIH guidelines. Animals were randomly assigned to experimental groups.

### AAA Models

Elastase model: AAA was induced by elastase exposure in mice at the age of 8 to 12 weeks as described previously.^[^ ^46^, ^47^ ^]^ After anesthesia, the aorta was isolated from the renal vein to the iliac bifurcation and was bathed in either 10 µL of 100% porcine pancreatic elastase (#E1250, Sigma‐Aldrich, St. Louis, MO) or heat‐inactivated elastase (control) for 40 min. After elastase exposure, the incision was stitched with a 4‐0 suture.

Calcium phosphate (CaPO_4_) model: AAA was induced by CaPO_4_ in mice at the age of 8 to 12 weeks as described previously.^[^ ^48^ ^]^ Briefly, mice were anaesthetized with ketamine (50 mg kg^−1^) and pentobarbital sodium (50 mg kg^−1^), and the infrarenal region of the abdominal aorta was isolated; then, a small piece of gauze soaked in 0.5 m CaCl_2_ was applied around the aorta. After 10 min, the gauze was replaced by another piece of phosphate‐buffered saline (PBS)‐soaked gauze for 5 min. Control mice were similarly treated with 0.5 m NaCl for 15 min. Sham‐operated mice were used as experimental controls.

Mice were sacrificed 7 days (CaPO_4_ model) or 14 days (elastase model) after the surgery, and the abdominal aorta was harvested and photographed (Nikon D7200) to determine its external diameter by using Image‐Pro Plus 6.0 computer‐assisted image analysis software. The adventitial circumferences at the maximal expanded portion of the infrarenal aortas were quantified as the maximal abdominal aortic diameter. At least 3 measurements of the maximal expanded portion of the infrarenal aorta of each mouse were averaged before calculating the mean of each experimental group. AAA was defined as an aortic diameter greater than 50% of the baseline measurement.

### Flow Cytometry Assay

Cells were isolated on ice, and single‐cell suspensions were prepared for use.^[^ ^49^ ^]^ To obtain single cells from an aorta, the whole aortas were minced into small pieces and then digested in 1X Aorta Dissociation Enzyme stock Solution (125 U mL^−1^ collagenase type XI, 60 U mL^−1^ hyaluronidase type I‐s, 60 U mL^−1^ DNase I, and 450 U mL^−1^ collagenase type I, all enzymes were obtained from Sigma‐Aldrich) at 37°C for 1 h. Cell suspensions were prepared by filtering them through a cell strainer (#322 350, 70 µm size, BD Bioscience, Billerica, MA). Cells were then stained by incubation with surface markers for 30 min on ice. For intracellular staining, cells were fixed and permeabilized using a Intracellular Fixation and Permeabilization buffer set (No. 00‐5523‐00, eBioscience, San Diego, CA) after surface marker staining, followed by staining with antibodies against intracellular antigens. For cell apoptosis detection, SMCs were collected, washed with PBS twice and stained with the annexin V and 7‐AAD according to the manufacturer's instructions. Stained cells were analyzed with a flow cytometer (FACS Aria III, BD). All reagents used for flow cytometry are listed in Table S1 (Supporting Information).

### Depletion and Expansion of Tregs

To deplete Tregs, DEREG mice were injected with Diphtheria Toxin (DT, Merck) diluted in endotoxin‐free PBS. Accordingly, DEREG mice were injected i.p. with 1 µg per mouse DT or PBS on day ‐2 and day ‐1 prior to AAA induction (performed on day 0), and then the process was repeated on day 7 and day 8.

Depletion of CD25^+^ cells was accomplished by i.p. injection of 200 µg of anti‐CD25 mAb (clone PC61, No. 102 040, Biolegend, San Diego, CA) or rat IgG (Biolegend) at days ‐3 and 8.

For expansion of Tregs, 5 µg of anti‐mouse IL‐2 mAb (503 702, Biolegend, San Diego, CA) and 1 µg of mouse IL‐2 (575 406, Biolegend, San Diego, CA) per mouse was incubated for 30 min at 37°C. Then, 8‐week‐old C57 mice were intraperitoneally injected with the IL‐2 complex for 3 consecutive days and thereafter every three days until day 14. Control mice were administered PBS.

### RNA‐Sequencing Analysis

Single‐cell suspensions were produced from the spleen, LNs or infrarenal aorta of AAA mice for Treg analysis, which was performed by flow cytometry. Approximately 1000 Tregs/Tconvs per three mice were collected and quickly placed into 6 µL of the SMART‐SeqTM v4 kit lysate supplied by the company. After measuring the volume of the fresh cell sample, a SMART‐SeqTM v4 UltraTM Low Input RNA Kit for Sequencing (Clontech) kit was used for cell lysis and synthesis of first‐strand cDNA. A total amount of 3 µg of RNA per sample was used as input material for RNA sample preparation. Sequencing libraries were generated using the NEBNext UltraTM RNA Library Prep Kit for Illumina (NEB, USA) following the manufacturer's recommendations, and index codes were added to attribute sequences to each sample. Briefly, mRNA was purified from total RNA using poly‐T oligo‐attached magnetic beads. Fragmentation was carried out using divalent cations under elevated temperature in NEBNext First Strand Synthesis Reaction Buffer (5X). First‐strand cDNA was synthesized using random hexamer primers and M‐MuLV Reverse Transcriptase (RNase H‐). Second‐strand cDNA synthesis was subsequently performed using DNA Polymerase I and RNase H. The remaining overhangs were converted into blunt ends via exonuclease/polymerase activities. After adenylation of the 3’ ends of DNA fragments, NEBNext adaptors with hairpin loop structures were ligated to prepare for hybridization. To preferentially select cDNA fragments 250–300 bp in length, the library fragments were purified with the AMPure XP system (Beckman Coulter, Beverly, USA). Then, 3 µL of USER Enzyme (NEB, USA) was used with size‐selected, adaptor‐ligated cDNA at 37°C for 15 min, followed by 5 min at 95°C before PCR. Then, PCR was performed with Phusion High‐Fidelity DNA polymerase, universal PCR primers and Index (X) Primer. Finally, the PCR products were purified (AMPure XP system), and library quality was assessed on the Agilent Bioanalyzer 2100 system.

Clustering of the index‐coded samples was performed on a cBot Cluster Generation System using the TruSeq PE Cluster Kit v3‐cBot‐HS (Illumina) according to the manufacturer's instructions. After cluster generation, the libraries were sequenced on an Illumina platform, and 125 bp/150 bp paired‐end reads were generated.

Differential expression analysis of two conditions/groups (two biological replicates per condition) was performed using the DESeq2 R package (1.1 6.1). DESeq2 provides statistical routines for determining differential expression in digital gene expression data using a model based on the negative binomial distribution. The resulting P values were adjusted using Benjamini and Hochberg's approach for controlling the false discovery rate. Genes with a *P* value <0.05, as determined by DESeq2, were deemed differentially expressed.

### Single‐Cell RNA‐Sequencing

Cell suspensions were produced from the spleens or infrarenal aortas from AAA mice for single cell analysis. Cell capture and cDNA synthesis were performed using a single‐cell 5′ Library and Gel Bead Kit (10x Genomics, 1000006) and Chromium Single Cell A Chip Kit (10x Genomics, 120236). The cell suspension (300–600 living cells per microliter determined by CountStar) was loaded onto the Chromium single‐cell controller (10x Genomics) to generate single‐cell gel beads in the emulsion according to the manufacturer's protocol. In short, single cells were suspended in PBS containing 0.04% BSA. Approximately 10 000 cells were added to each channel, and the target cells recovered were estimated to be approximately 6000 cells. Captured cells were lysed, and the released RNA was barcoded through reverse transcription in individual GEMs.

Reverse transcription was performed on a S1000TM Touch Thermal Cycler (Bio Rad) at 53°C for 45 min, followed by 85°C for 5 min, and a final hold of 4°C. cDNA was generated and then amplified, and quality was assessed using an Agilent 4200 (performed by CapitalBio, Beijing).

According to the manufacturer's instructions, single‐cell RNA‐seq libraries were constructed by using the Single Cell 5’ Library and Gel Bead Kit, Single Cell V(D)J Enrichment Kit, mouse T Cell (1000071) and Single Cell V(D)J Enrichment Kit. The libraries were finally sequenced using an Illumina NovaSeq6000 sequencer with a sequencing depth of at least 100 000 reads per cell with a paired‐end 150 bp (PE150) reading strategy (performed by CapitalBio, Beijing).

For single‐cell TCR‐sequencing, only cells with both productive TCR*α* and TCR*β* chains were used for analysis. For cells which were defined as the same clonotype, they were expected to share both TCR*α* and TCR*β* sequences. The number (two or more) of cells with such TCR*α*‐TCR*β* pairs indicated the degree of clonality of the clonotype and the ratio of clonal expansion was the proportion of cells with the same clonotype among all cells analyzed.

Gini coefficient was calculated within individuals and then averaged for all mice, the Gini coefficient defines the distribution of clonotype used for each epitope. This provides a measure of inequality, based on a curve plotting the cumulative proportion of total sequences on the *y* axis relative to the individual clonotype use on the *x* axis, with the coefficient being calculated using the ineq package in R. The distribution of sequence abundances and repertoire evenness were evaluated using the Gini inequality coefficient, which ranges from 0 for a repertoire where every sequence is present in equal abundance, to 1 for a repertoire dominated by a single sequence, with other sequences present at zero abundance.^[^ ^50^ ^]^

### FTY720 and IL‐33 Treatment

To block the exit of lymphocytes from peripheral lymphoid organs, mice were treated with an S1P1 receptor agonist. FTY720 (1 mg kg^−1^) (No. SML0700, Sigma‐Aldrich, Merck, Germany) was i.p. injected one day prior to injury and daily thereafter.

To observe the maintenance of IL‐33 on aorta Tregs, mice were i.p. injected with recombinant IL‐33 (No. 34‐8332‐85, eBioscience, San Diego, CA) at 1 µg per injection daily in the CaPO_4_ model for 7 days and in the elastase model for 14 days.

### Parabiosis Experiments

Mice were joined in parabiosis as described previously.^[^ ^23^ ^]^ B6 CD45.1 and B6 CD45.2 mice were conjoined for 2 weeks. Mice received buprenorphine (0.1 mg kg^−1^ i.p.) twice daily for 3 days, starting on the day of the surgery for 2 weeks. Experiments began 14 days after parabiosis surgery, as required to establish a shared circulation. Then, a PPE‐induced surgery was conducted in the CD45.1 parabiont and analyzed the chimerism of CD45.2^+^ Tregs in the blood and aorta 2 weeks later.

To calculate the contribution of recruitment to the expansion of Tregs in AAA, data obtained in the parabiosis experiment shown in Figure 4e was used. The chimerism of CD45.2^+^ cells in the blood and aorta in the CD45.1 parabionts (Figure 4e) was first assessed. Then amount of recruitment was calculated by normalizing the aorta‐to‐blood chimerism for each mouse.^[^ ^23^ ^]^ For instance, if the blood chimerism was 35.7% and the aorta chimerism was 30.9%, recruitment equaled 86.6% after normalization to blood: 100%/35.7%×30.9% = 86.6%. Two weeks after AAA induction, 5375 Tregs/aorta was counted by flow cytometry, which increased from 20 in the steady state (Figure 1c). Thus, an additional 5355 Tregs accumulated in the aorta. The parabiosis experiment and the above calculation determined that an average of 78.6% of the 5355 Tregs originated from recruitment (on average, 4209 CD45.2^+^ Tregs accumulated in CD45.1 parabiont aorta, experiment outlined in Figure 4e). Then these 4209 Tregs was normalized to the mean number of Tregs that were produced by AAA induction (4209/5375 * 100% = 78.3%), which indicated that 78.3% of the Treg increase was driven by recruitment (Figure 4e).

### Migration Studies Using KikGR/B6 Mice

Mice were anesthetized, and the fur attached to the skin covering the CLNs was removed using depilatory cream. Mice were placed on their backs with an aluminum foil blanket covering all but the depilated area. Violet light (Electra Pro Series 405 nm Violet Handheld Laser, Changchunxinjian Technologies, China) was shone on the exposed area for a period of 3.5 min, which is the pre‐established time for effective but innocuous photoconversion at this anatomical site. To manipulate the size of the light field (beam diameter: 3.5 mm) so that both CLNs could be exposed, a lens was attached to the laser to defocus the beam, and the source of the defocused light beam was positioned 28 cm above the mouse.^[^ ^51^ ^]^

### Tconvs Adoptive Transfer

To perform Tconv adoptive transfer in CD45.1 mice, Tconvs was sorted from CD45.2 Foxp3‐GFP mice. The CD4^+^GFP^–^ Tconvs were sorted by flow cytometry, and 5 × 10^6^ Foxp3^–^CD4^+^ Tconvs were iv‐transferred into 8‐week‐old CD45.1 recipient mice.

To perform Treg adoptive transfer in DEREG mice, CD4^+^CD25^+^Tregs was sorted from WT and Tff1^–/–^ mice. The CD4^+^CD25^+^Tregs were sorted by magnetic‐activated cell sorting and 2 × 10^6^ CD4^+^CD25^+^Tregs were iv‐transferred into 8–12 week‐old DEREG recipient mice.

### Quantitative PCR

Aorta tissue was frozen in liquid N_2_ and homogenized in Trizol (Invitrogen) before RNA extraction according to manufacturer's instructions. RNA was reverse transcribed with oligo (dT) primers and SuperScript Polymerase 2 (Invitrogen). Real‐time quantitative PCR was performed using gene‐specific fluorogenic assays (TaqMan; Applied Biosystems). Transcript levels were normalized to expression of mouse 18S gene. The primers used in the article are listed as follows: Tff1, Forward: TAAATTGTGGCTTCCCCGGT, Reverse: AGGGACATTCTTCTTCTTGAGTGT; and 18S, Forward: CCTGGATACCGCAGCTAGGAA, Reverse: TTTCGCTCTGGTCCGTCTTG.

### Histology and Immunohistochemical (IHC) Analyses

After the mice were sacrificed, aortas from the ascending aorta to the bifurcation of the iliac artery were isolated. Infrarenal aortas were harvested, fixed for 24 h in 4% paraformaldehyde, cut at the site of maximal diameter, and embedded in paraffin for cross section preparation.

For immunofluorescence staining, paraffin sections (4 µm each) were permeabilized with 0.3% Triton X‐100 in PBS for 5 min and blocked with 5% fetal bovine serum and 10% donkey serum in PBS at room temperature for 1 h. Tissues were stained for 2 h at room temperature in the presence of rabbit anti‐*α*‐ smooth muscle actin (*α*‐SMA), followed by staining for 1 h at room temperature with a PE‐donkey anti‐rabbit secondary antibody. Apoptotic cells in lesions were determined with the TUNEL apoptosis detection kit according to the manufacturer's instructions (#ab206386, Abcam). Tissue sections were further stained with DAPI for 5 min before imaging (Nikon A1Si). Five mice were randomly selected from each experimental group for immunohistological analysis based on the availability. All examinations were conducted by two trained, independent observers who were blinded to the genotype and treatment. A mean value was determined from four sections from each animal.

### Isolation and Culture of Primary VSMCs

Primary mouse VSMCs were isolated from the aorta of WT mice aged 8 weeks and cultured as described previously.^[^ ^52^ ^]^ The cells were used in all experiments from passages 4–7. The cells with a density at 80–90% were treated with Tff1 (500 ng mL^−1^, PeproTech) and TNF‐*α* (100 ng mL^−1^) for 24 h. Then, after 24 h of incubation apoptosis was detected with an Annexin V detection kit according to the manufacturer's instructions (eBioscience). To test the Tff1 ERK‐dependent role, VSMCs were co‐cultured with Tff1 and ERK inhibitor (10 × 10^−6^ m, ERK1/2 inhibitor 1, Selleck, SCH77298), and VSMCs were pretreated with ERK inhibitor for 1 h before the exposure to Tff1 for 24 h.

### Adenovirus Transfection

For in vivo studies, 2×10^9^ pfu of adenovirus carrying Tff1 was dissolved in 200 µL of enhanced transfection solution and was injected through the tail vein. The control group was given an equal volume of blank‐transfected adenovirus. All viral transfections were performed on the day the PPE model was established.

### Western Blot

Equal amounts of total protein from cells were loaded onto a 10% SDS (sodium dodecyl sulfate) polyacrylamide gels. Proteins were transferred on PVDF membranes using a Trans‐Blot Turbo Transfer System (Bio‐Rad). Membranes were blocked and probed for ERK1/2 (1:1000 dilution; cat# 4695, Cell Signaling Technology), pERK1/2 (1:1000 dilution; cat# 4370, Cell Signaling Technology), Caspase‐3 (1:1000 dilution; cat# 9662; Cell Signaling Technology), Cleaved‐caspase‐3 (1:1000 dilution; cat# 9664; Cell Signaling Technology), Caspase‐8 (1:1000 dilution; cat# 4927; Cell Signaling Technology), Cleaved‐caspase‐8 (1:1000 dilution; cat# 9429; Cell Signaling Technology), Caspase‐9 (1:1000 dilution; cat# 9504; Cell Signaling Technology), Cleaved‐caspase‐9 (1:1000 dilution; cat# 9509; Cell Signaling Technology), and GAPDH (1:1000 dilution; ab8245, Abcam). HRP‐conjugated species‐appropriate secondary antibodies (1:3000) were applied before bands were visualized with the ECL reagent (Pierce, Rockford, IL). GAPDH was used as a control. Immunoblot signals were detected by enhanced chemiluminescence with ChemiDoc XRS (Bio‐Rad Laboratories).

### Statistical Analysis

Data are presented as the mean ± S.E.M. To assess statistical significance, the Shapiro‐Wilk normality test was first employed to determine if the data were distributed normally. The variance between groups was tested by the F test. If evaluation revealed similar variances, then Student's *t* test was used for comparisons between two groups. A nonparametric Mann‐Whitney U test was used when the data were not normally distributed. For comparisons among three or more groups, if passed tests of normal distribution, one or two‐way ANOVA and a post hoc Bonferroni test were used. A nonparametric Kruskal‐Wallis test was used when the data were not normally distributed. No data or outliers were excluded. In all cases, statistical significance was indicated when the two‐tailed probability was less than 0.05. Sample size is provided in each figure legend. All analyses were performed using GraphPad Prism 7.0 and SPSS 18.0 software.

## Conflict of Interest

The authors declare no conflict of interest.

## Author Contributions

J.L., N.X., and D.L. contributed equally to this work. J.L. designed and performed the experiments, analyzed the data, and wrote the paper; N.X. organized, designed, and wrote the paper; D.L. performed the experiments and analyzed the data; S.W., S.Q., Y.L., and M.G. performed the experiments; T.T., J.J., B.L., S.N., D.H., Y.L., X.Y., and G.S. organized and designed the experiments; and X.C. initiated the study and organized, designed, and wrote the paper.

## Supporting information

Supporting Information

## Data Availability

The data that support the findings of this study are available from the corresponding author upon reasonable request.
