# Supplementary material for: Aorta Regulatory T Cells with a Tissue‐Specific Phenotype and Function Promote Tissue Repair through Tff1 in Abdominal Aortic Aneurysms
Source: Adv Sci (Weinh). 2022 Jan 24;9(9):2104338. doi: 10.1002/advs.202104338 (PMC8948580; doi:10.1002/advs.202104338)
Supplement: Supplementary file 1 — Supporting Information [file ADVS-9-2104338-s001.pdf]

ADVANCED SCIENCE
Open Access

Supporting Information

for *Adv. Sci.*, DOI 10.1002/advs.202104338

Aorta Regulatory T Cells with a Tissue-Specific Phenotype and Function Promote Tissue Repair through Tff1 in Abdominal Aortic Aneurysms

*Jingyong Li, Ni Xia, Dan Li, Shuang Wen, Shirui Qian, Yuzhi Lu, Muyang Gu, Tingting Tang, Jiao Jiao, Bingjie Lv, Shaofang Nie, Desheng Hu, Yuhua Liao, Xiangping Yang, Guoping Shi and Xiang Cheng**

ADVANCED SCIENCE
Open Access

Supporting Information

for *Adv. Sci.*, DOI: 10.1002/advs.202104338

Aorta regulatory T cells with a tissue-specific phenotype and function promote tissue repair through Tff1 in abdominal aortic aneurysms

*Jingyong Li, Ni Xia, Dan Li, Shuang Wen, Shirui Qian, Yuzhi Lu, Muyang Gu, Tingting Tang, Jiao Jiao, Bingjie Lv, Shaofang Nie, Desheng Hu, Yuhua Liao, Xiangping Yang, Guoping Shi, and Xiang Cheng**

Supplemental Materials

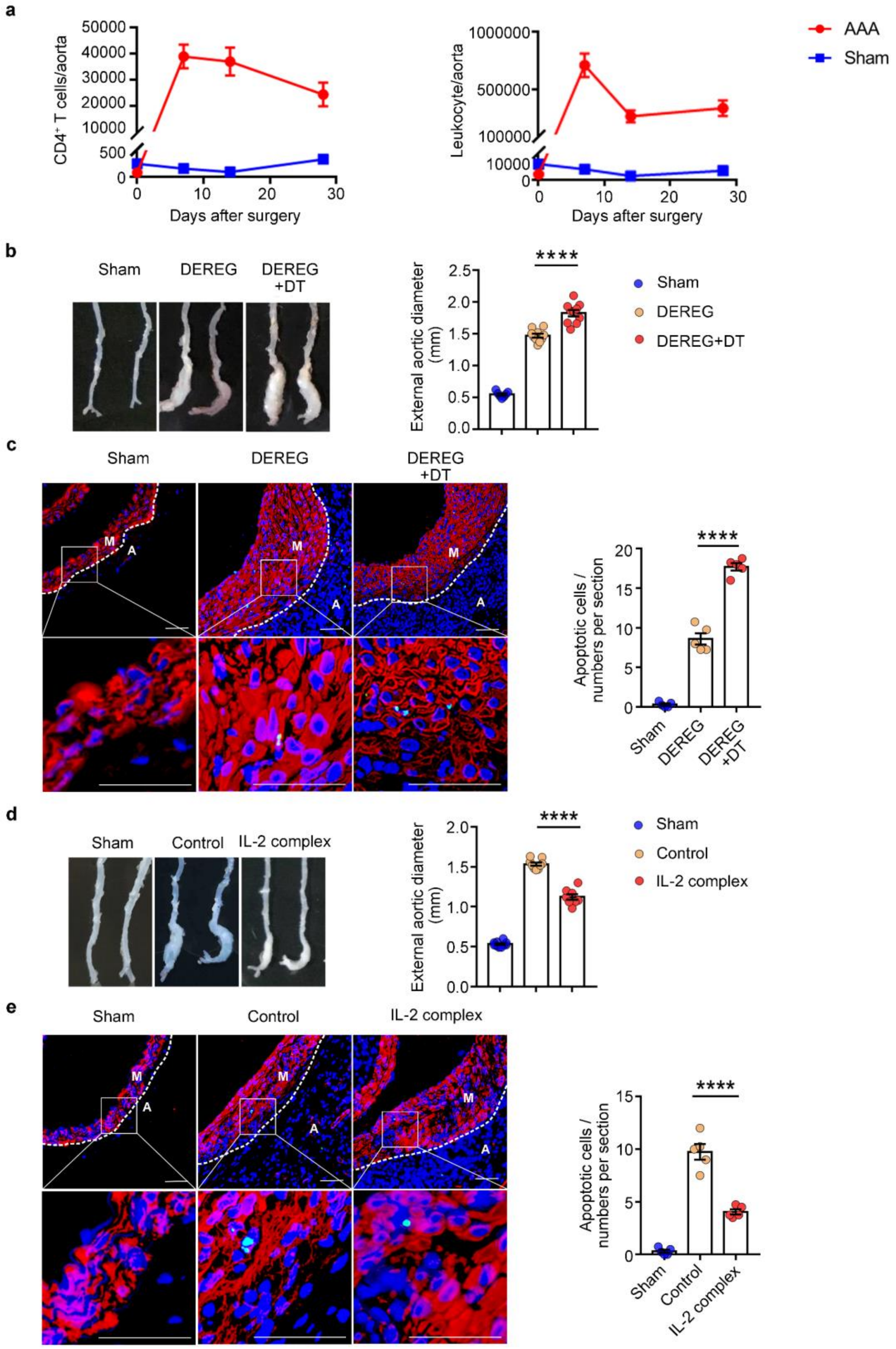

Figure S1. Inflammatory cells infiltrate the aorta during the AAA process and the role of Tregs in AAA

(a) Numbers of different subgroups of inflammatory cells in the aorta of AAA mice at

different time points (Day 0, 7, 14, 28, n=4-5 per group). (b) Representative photographs of abdominal aortic fragments from the 3 groups of mice and quantification of the maximal external diameter of the infrarenal aortas (Sham=7, DEREG=10, and DEREG+DT=10), one-way ANOVA, ****$p<0.0001$. (c) Representative immunofluorescent image of SMC apoptosis from the 3 groups of mice and quantification of the numbers of TUNEL-positive SMCs (n=5 per group), one-way ANOVA, ****$p<0.0001$. (d) Representative photographs of abdominal aortic fragments from the 3 groups of mice and quantification of the maximal external diameter of the infrarenal aortas (Sham=10, Control=9, IL-2 complex=8), one-way ANOVA, ****$p<0.0001$. (e) Representative immunofluorescent image of SMC apoptosis from the 3 groups of mice and quantification of the numbers of TUNEL-positive SMCs (n=5 per group), one-way ANOVA, ****$p<0.0001$. Data are presented as mean ± S.E.M.

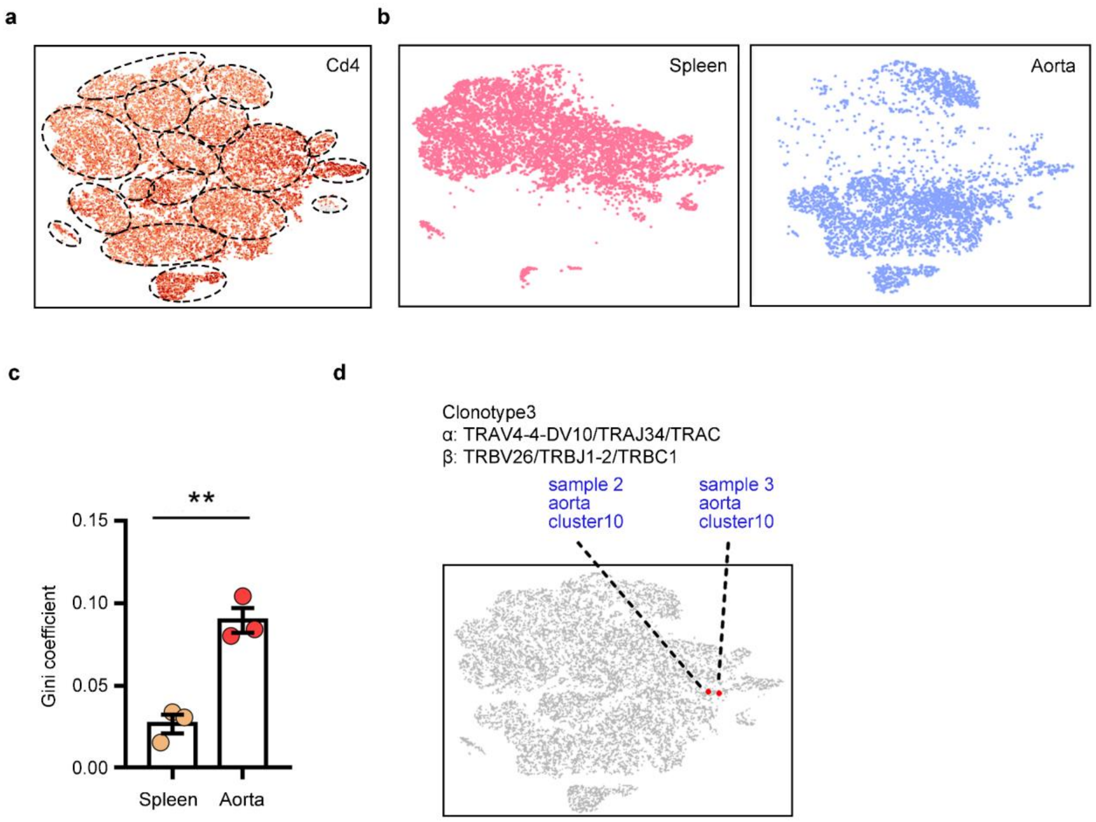

Figure S2. Single-cell transcriptome and TCR repertoires analysis of aorta Tregs

(a) t-SNE plot of the expression levels of *Cd4* in different clusters. (b) t-SNE plot of the distribution of all cells in the spleen and aorta. (c) The repertoire evenness was assessed by the Gini coefficient (n=3 per group), a Gini coefficient value of 0 denotes an even repertoire, and a value of 1 denotes a repertoire dominated by a single sequence, unpaired 2-tailed t test, **$p<0.01$. (d) The distribution of representative Treg clones on the t-SNE plot between different samples. Data are presented as mean ± S.E.M.

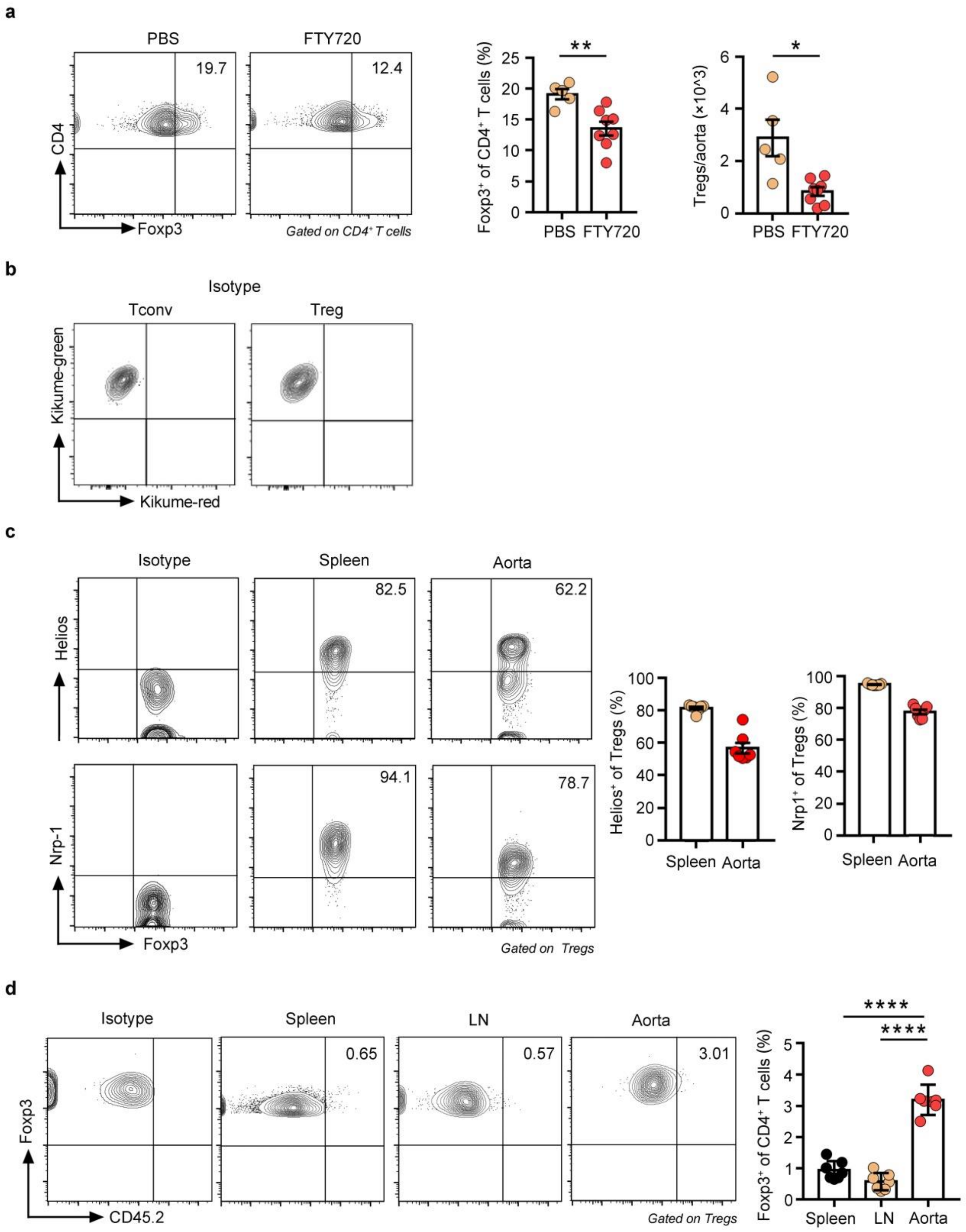

Figure S3. The origin of aorta Tregs

(a) Two-month-old mice were treated with PBS or FTY720 one day prior to PPE-induced AAA and daily thereafter. Aorta Tregs were analyzed by flow cytometry analysis. Representative FACS images are shown on the left and quantification of the percentages is shown on the right (PBS=5, FTY720=8), unpaired 2-tailed t test, *p<0.05, **p<0.01. (b) Representative FACS images of Kikume red^{+} cells with no

photoconversion in the aorta pregated for $CD4^{+}CD25^{-}$ Tconvs (left) or $CD4^{+}CD25^{+}$ Tregs (right). (c) Flow cytometry analysis of Helios and Nrp-1 expression in spleen and aorta Tregs. Representative FACS images are on the left, and quantification of the percentages is on the right (n=7 per group). (d) $Foxp3^{-}$Tconvs from pooled spleens of $CD45.2^{+}$Foxp3-GFP donors were iv-transferred into $CD45.1^{+}$ recipients and analyzed 2 weeks later. Quantification of the percentages of Tregs induced from Tconvs is shown. Representative FACS images are on the left, and quantification of the percentages is on the right (n=7 per group). The results are a combination of three independent experiments, one-way ANOVA, ****$p<0.0001$. Data are presented as mean ± S.E.M.

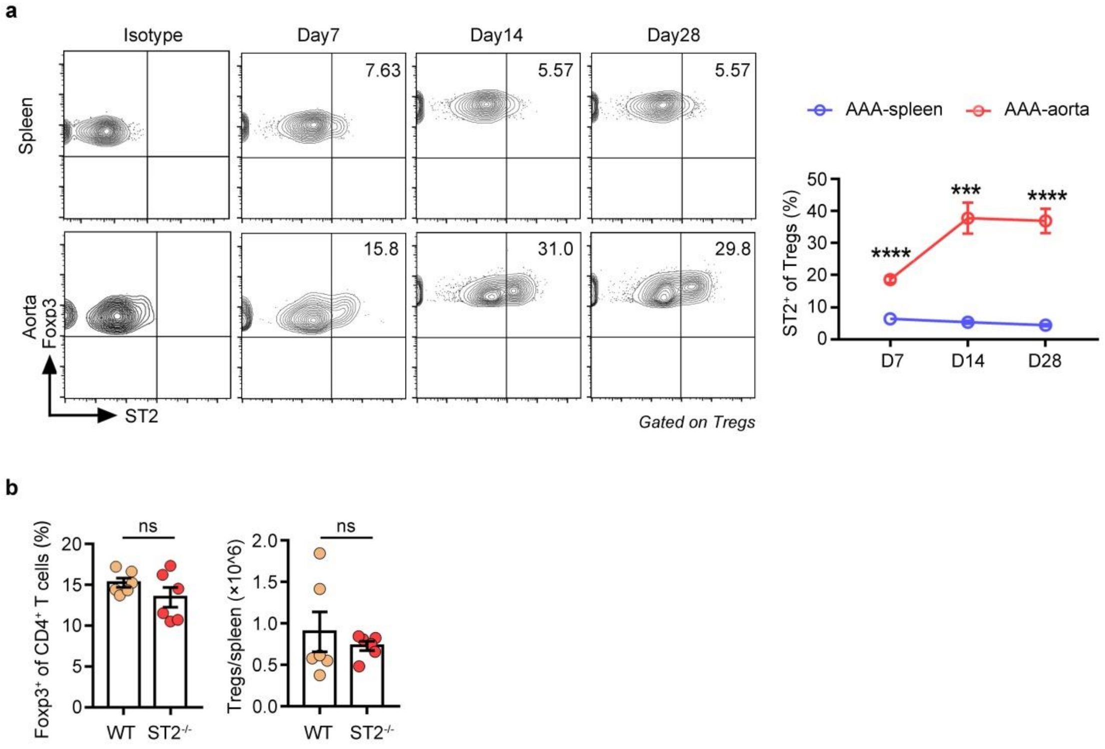

Figure S4. Involvement of the IL-33/ST2 axis in aorta Treg maintenance

(a) Flow cytometry analysis of ST2 expression in spleen and aorta Tregs in AAA mice at different time points. Representative FACS images are on the left, and quantification of the percentages is on the right (n=4-6 per group), unpaired 2-tailed t test, ***p<0.001, ****p<0.0001. (b) Percentages and numbers of spleen Tregs in WT and $ST2^{-/-}$ mice 14 days after surgery (n=6 per group), unpaired 2-tailed t test, ns: not significant. Data are presented as mean ± S.E.M.

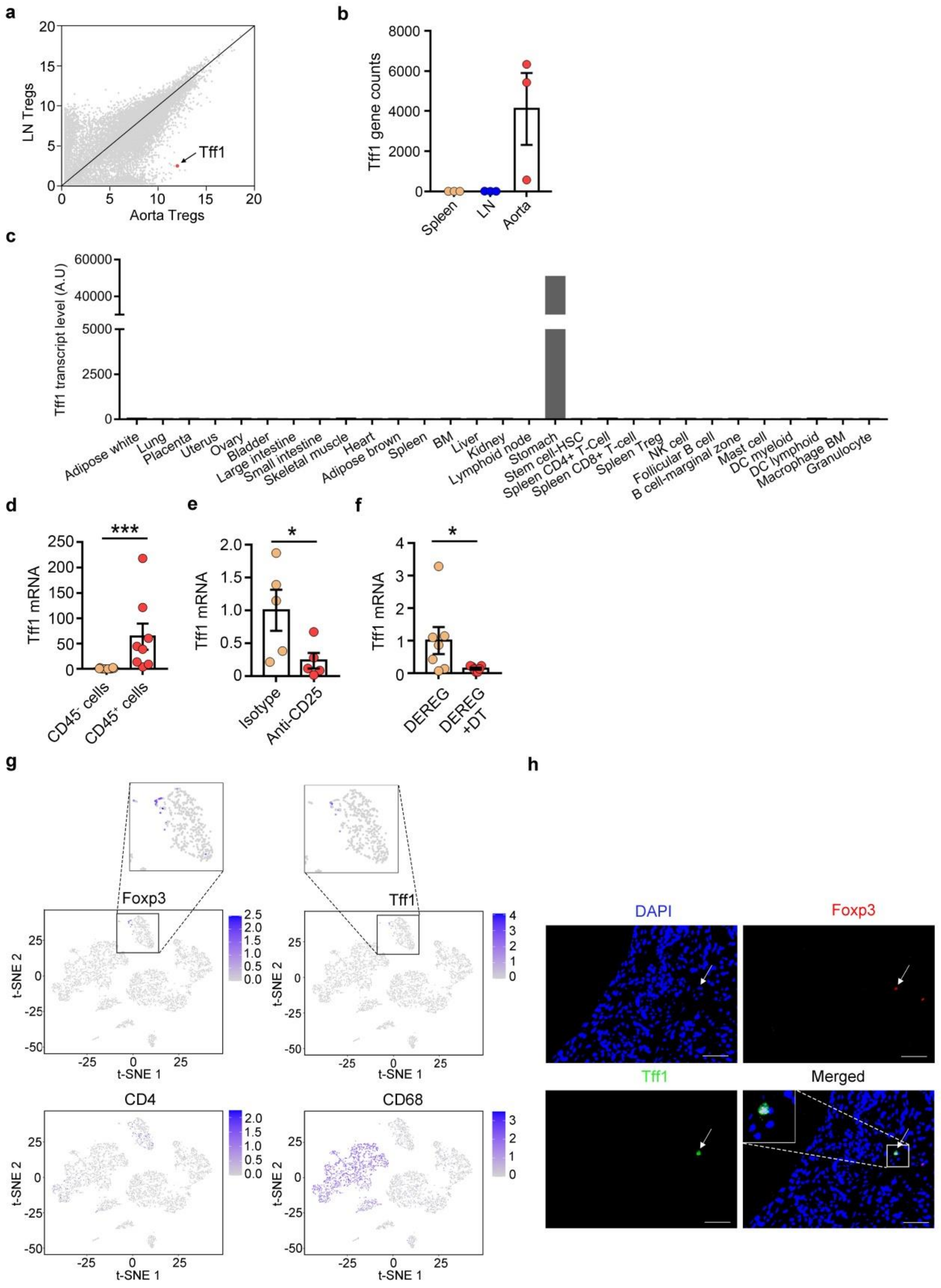

Figure S5. Aorta Tregs specifically express Tff1

(a) Scatter plots comparing gene expression quantified by RNA sequencing of aorta Tregs vs. LN Tregs. The Tff1 gene is highlighted in red. (b) Raw gene counts of the Tff1 transcript were quantified by RNA sequencing. (c) Expression of Tff1 in BioGPS

RNA-seq data sets from different tissues (GeneAtlas MOE430, gcrma); AU: arbitrary units. (d) RT-PCR analysis of Tff1 mRNA levels in $CD45^-$ and $CD45^+$ fractions from digested AAA aortas 14 days after AAA induction (n=8 per group) is shown, Mann-Whitney U test, ***p<0.001. (e) RT-PCR analysis of Tff1 mRNA levels in aortas from isotype- or anti-CD25-treated AAA mice 14 days after AAA induction (n=5 per group), unpaired 2-tailed t test, *p<0.05. (f) RT-PCR analysis of Tff1 mRNA levels in AAA aortas from PBS- or DT-treated DEREG mice 14 days after AAA induction (n=7 per group). The results are a combination of two independent experiments, Mann-Whitney U test, *p<0.05. (g) scRNA-Seq datasets from murine (Zhao et al. 2021) aneurysm lesions were analyzed and tSNE plots show the expression of Foxp3, Tff1, CD4 and CD68. (h) Immunofluorescent costaining of Tff1 (green) with Tregs (Foxp3: red) in mouse AAA tissues. Scale bar: 50 μm. Data are presented as mean ± S.E.M.

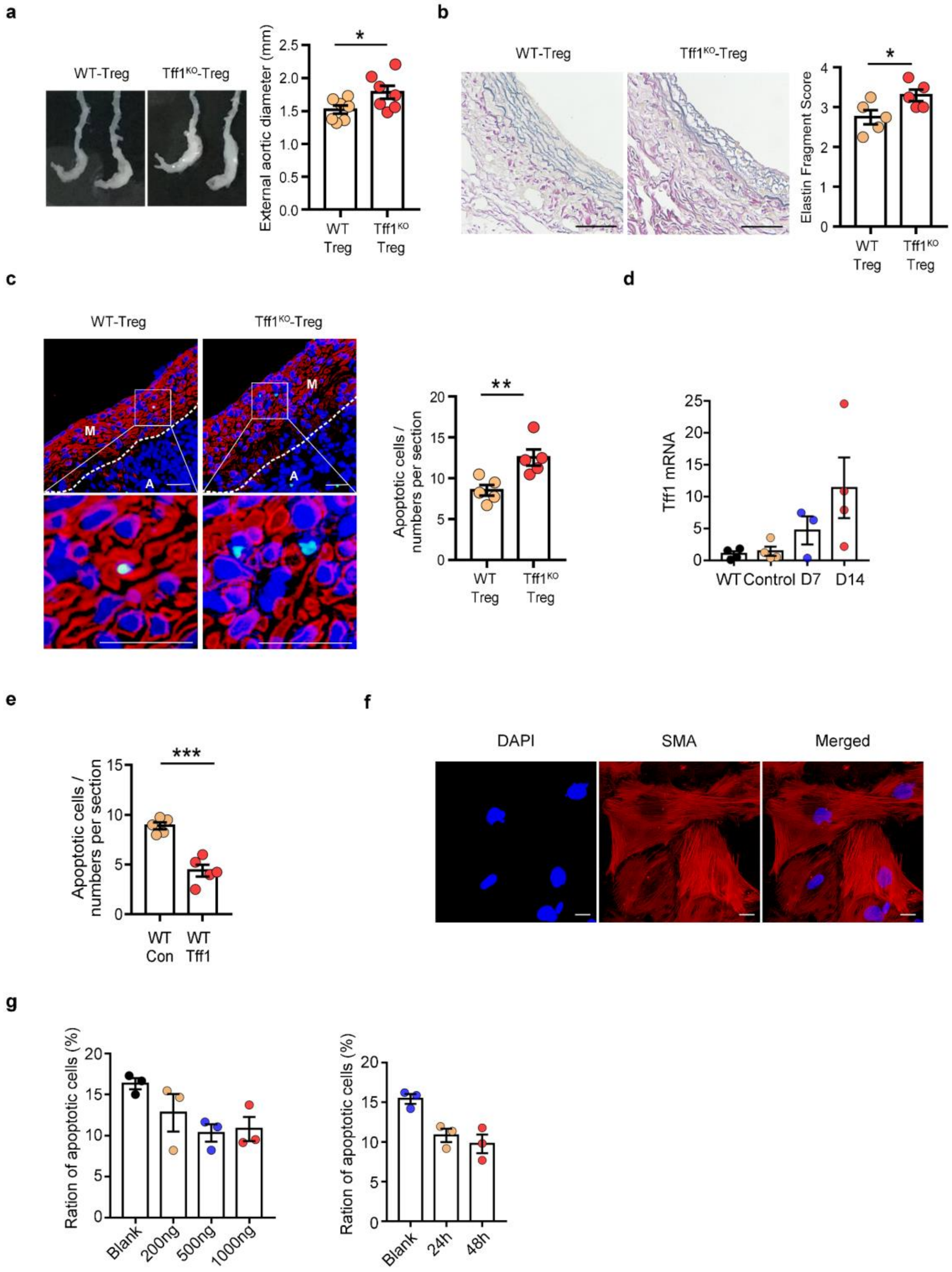

Figure S6. The role of Tff1 derived from Tregs in AAA

(a-c) DEREG mice were subjected to PPE-induced AAA and injected i.p. with 1 μg/mouse DT on day -2 and day -1 prior to AAA induction (performed on day 0), and then the process was repeated on day 7 and day 8. $CD4^{+}CD25^{+}$Tregs from WT and

$Tff1^{KO}$ mice were sorted by magnetic-activated cell sorting and 2 × 10^6 Tregs were iv-transferred into 8-12 week-old DEREG recipient mice. (a) Representative photographs of aortic fragments from the 2 groups: DEREG + DT + WT-Tregs (n=7), DEREG + DT + $Tff1^{KO}$-Tregs (n=7) and quantification of the maximal external diameter of the infrarenal aortas, unpaired 2-tailed t test, *p<0.05. (b) Representative images of elastic Verhoeff-Van Gieson (EVG) staining of aortic tissue from the 2 groups of mice and the assessment of medial elastica fragmentation (n=5 per group), unpaired 2-tailed t test, *p<0.05, scale bar: 50 μm. (c) Representative immunofluorescent image of SMC apoptosis from the 2 groups of mice. Left: α-SMA (red) is costained with TUNEL (green). Right: Quantification of the numbers of apoptotic SMCs (n=5 per group), unpaired 2-tailed t test, **p<0.01, scale bar: 50 μm. M: media, A: adventitia. (d) RT-PCR analysis of Tff1 mRNA levels in aortas from control adenovirus- or Tff1 adenovirus-treated mice, (n=3-4 per group). WT group: 14 days after AAA induction with no adenovirus; Control group: 14 days after AAA induction with control adenovirus; D7 group: 7 days after AAA induction with Tff1 adenovirus; D14 group: 14 days after AAA induction with Tff1 adenovirus. (e) Quantification of the numbers of TUNEL-positive SMCs (n=5 per group), unpaired 2-tailed t test, ***p<0.001. (f) Mouse primary VSMCs were stained with antibody against α-SMA as a SMC maker (red), scale bar: 20μm. (g) Left: Different concentrations (200 ng/ml, 500 ng/ml, and 1000 ng/ml) of Tff1 were used to treat the VSMCs, and apoptosis was detected by flow cytometry 24 h later. Blank: no treatment with 24 h; Right: 500 ng/ml Tff1 was applied to the VSMCs, and apoptosis was detected by flow cytometry at different time points (24 h, 48 h), blank: no treatment with 48 h, n=3 per group. Data are presented as mean ± S.E.M.

Supplementary Table I. Antibodies used in this article

**FACS antibodies**	**Dilutions**	**Concentrations**	**Catalog numbers**	**Company names and addresses**
FITC-anti-CD45	1:200	2.5 μg/ml	103108	BioLegend,

				San Diego, CA
APC/CY7-anti-CD45	1:80	2.5 µg/ml	103116	BioLegend, San Diego, CA
BV510-FVD	1:100	Not available	65-0866-14	eBioscience, San Diego, CA
BV421-anti-F4/80	1:80	2.5 µg/ml	123137	BioLegend, San Diego, CA
APC-anti-F4/80	1:80	2.5 µg/ml	123116	BioLegend, San Diego, CA
PE/CY7-anti-CD11b	1:80	2.5 µg/ml	101216	BioLegend, San Diego, CA
PerCP-CY5-5-anti-CD11b	1:80	2.5 µg/ml	101228	BioLegend, San Diego, CA
PE/CY7-anti-CD4	1:80	2.5 µg/ml	100422	BioLegend, San Diego, CA
APC-anti-CD4	1:80	2.5 µg/ml	100412	BioLegend, San Diego, CA
PE-anti-CD4	1:80	2.5 µg/ml	100408	BioLegend, San Diego, CA
PE-anti-Foxp3	1:20	10 µg/ml	126404	BioLegend, San Diego, CA
PE/CY7-anti-Nrp-1	1:80	2.5 µg/ml	145212	BioLegend,

				San Diego, CA
APC/CY7-anti-Helios	1:20	0.15 μg/ml	47-9883-42	eBioscience, San Diego, CA
PerCP-CY5-5-anti-ST2	1:80	2.5 μg/ml	145312	BioLegend, San Diego, CA
APC-anti-ST2	1:80	2.5 μg/ml	146606	BioLegend, San Diego, CA
APC-anti-Ki67	1:50	10 μg/ml	652418	BioLegend, San Diego, CA
FITC-anti-Ki67	1:200	2.5 μg/ml	151204	BioLegend, San Diego, CA
PerCP-CY5-5-7AAD	1:20	2.5 μg/ml	559925	BD Pharmingen ™
APC-anti-CD25	1:80	2.5 μg/ml	101910	BioLegend, San Diego, CA
**Immunofluorescent staining antibodies**	**Dilutions**	**Concentrations**	**Catalog numbers**	**Company names and addresses**
α-SMA	1:100	2 μg/ml	ab5694	Abcam, Cambridge, MA
PE-donkey anti-goat secondary antibody	1:100	5 μg/ml	ab98520	Abcam, Cambridge, MA
Tff1	1:100	2 μg/ml	ab92377	Abcam,

				Cambridge, MA
DyLight® 488-Goat Anti-Rabbit secondary antibody	1:250	2 μg/ml	ab96899	Abcam, Cambridge, MA
Anti-Foxp3	1:25	NA	320114	BioLegend, San Diego, CA
